# Diterpenoids from the Brown Alga *Rugulopteryx okamurae* and Their Anti-Inflammatory Activity

**DOI:** 10.3390/md19120677

**Published:** 2021-11-27

**Authors:** Belén Cuevas, Ana I. Arroba, Carolina de los Reyes, Laura Gómez-Jaramillo, M. Carmen González-Montelongo, Eva Zubía

**Affiliations:** 1 Departamento de Química Orgánica, Facultad de Ciencias del Mar y Ambientales, Universidad de Cádiz, 11510 Puerto Real (Cádiz), Spain; belen.cuevas@inibica.es (B.C.); carolina.dereyes@uca.es (C.d.l.R.); 2 Unidad de Investigación, Instituto de Investigación e Innovación Biomédica de Cádiz (INiBICA), Hospital Universitario Puerta del Mar, Avda. Ana de Viya 21, 11009 Cádiz, Spain; anaarroba@gmail.com (A.I.A.); laugomjar@gmail.com (L.G.-J.); mcmontel@gmail.com (M.C.G.-M.); 3 Departamento de Endocrinología y Nutrición, Hospital Universitario Puerta del Mar, Avda. Ana de Viya 21, 11009 Cádiz, Spain

**Keywords:** diterpenoids, brown algae, *Rugulopteryx okamurae*, anti-inflammatory, nitric oxide, cytokine, microglia, macrophages, invasive algae

## Abstract

Brown algae of the Family Dictyotaceae produce an array of structurally diverse terpenoids, whose biomedical potential in the anti-inflammatory area has been scarcely explored. Herein, the chemical study of the alga *Rugulopteryx okamurae* has led to the isolation of ten new diterpenoids: rugukadiol A (**1**), rugukamurals A–C (**2**–**4**), and ruguloptones A–F (**6**–**10**). The structures of the new compounds were established by spectroscopic means. Compound **1** exhibits an unprecedented diterpenoid skeleton featuring a bridged tricyclic undecane system. Compounds **2**–**10** belong to the secospatane class of diterpenoids and differ by the oxygenated functions that they contain. In anti-inflammatory assays, the new diterpenoid **1** and the secospatanes **5** and **10** significantly inhibited the production of the inflammatory mediator NO in LPS-stimulated microglial cells Bv.2 and macrophage cells RAW 264.7. Moreover, compounds **1** and **5** were found to strongly inhibit the expression of *Nos2* and the pro-inflammatory cytokine *Il1b* in both immune cell lines.

## 1. Introduction

Algae of the Family Dictyotaceae are a prolific source of natural products, which account for almost 40% of the metabolites isolated from brown algae [1]. Most of the isolated compounds are terpenoids, including sesquiterpenoids, diterpenoids, and meroterpenoids [1,2,3,4]. In particular, species of the genera *Dictyota*, *Canistrocarpus*, *Stoechospermum*, *Spatoglosum* and *Rugulopteryx*, are characterized by producing a wide series of cyclic diterpenoids. These metabolites display a variety of carbon skeletons, which differ significantly among genera and may be useful chemotaxonomic markers [2,3,4]. From the biomedical point of view, properties such as antimicrobial [5,6,7] and cytotoxic [8,9,10] activities of some diterpenoids were already described during the first studies of this family of algae, and more recently antiviral [11,12,13], antileishmaniosis [14], antithrombotic [15], and further antibacterial [16] and anticancer activities [16,17] have been reported.

Currently, there is a growing interest in the search for new anti-inflammatory agents, provided the key role that inflammation plays in the development of multiple diseases such as some types of cancer, rheumatoid arthritis, inflammatory bowel disease or diabetes, among others [18,19,20,21]. In this regard, data on the anti-inflammatory potential of diterpenoids from Dictyotacean algae are scarce and, only recently, a few metabolites of dolastane and xenicane types, isolated from *Dictyota plectens*, have shown anti-inflammatory properties [22,23].

As a part of our research project aimed to study new anti-inflammatory compounds from algae, we have examined specimens of the brown alga *Rugulopteryx okamurae* (Dictyotaceae) collected in the Strait of Gibraltar. *R. okamurae*, first known as *Dilophus okamurae* [24], is a species native to the northwestern Pacific Ocean, and can be found widely distributed along the coasts of China, Japan, Korea, Philippines, and Taiwan [25]. In recent years, *R. okamurae* has invaded the southwestern coasts of Europe, in particular the coasts of the Strait of Gibraltar [26]. The alga has experienced a proliferation so quick and massive that in a few years it has become a dominant species, which covers large areas of ocean bottom and produces tons of beach cast material [26,27].

Previous studies of *R. okamurae* collected at different locations of the Japanese coasts led to the isolation of more than twenty diterpenoids which display several carbon skeletons [28,29,30,31,32,33,34,35]. Recently, the chemical study of *R. okamurae* from the Spanish coasts has led to the isolation of six diterpenoids of secospatane, spatane, and prenylcubebane types, already reported from Japanese specimens of the alga [36]. Bioactivity data on terpenoids from *R. okamurae* are mostly focused on their ecological role as feeding deterrents of predators [29,30,32,35,36]. However, data on the biomedical potential are scarce, and only antibacterial activity of some secospatanes against *Bacillus subtilis* has been reported [33].

Herein we describe a reinvestigation of the extract of *R. okamurae* that has led to the isolation of ten new diterpenoids: rugukadiol A (**1**), rugukamurals A–C (**2**–**4**), and ruguloptones A–F (**5**–**10**). The known compounds dilkamural (**11**) and **12**, which are the major metabolites of the extract, were also obtained. The isolated compounds were tested in anti-inflammatory assays aimed to detect the inhibition of the production of the inflammatory mediator nitric oxide (NO) and the expression of *Nos2* and pro-inflammatory cytokines.

## 2. Results and Discussion

### 2.1. Isolation and Structure Determination 

**Figure 1:**
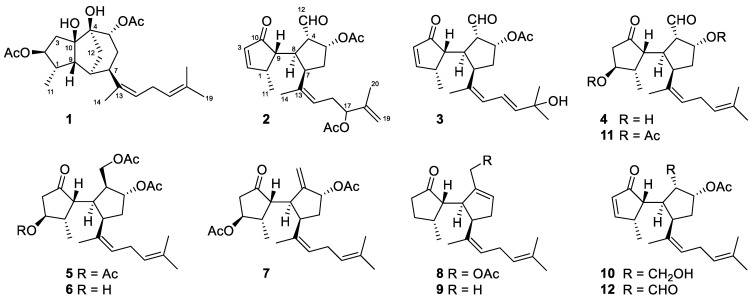
Chemical structures of the diterpenoids isolated from *R. okamurae*.

Fresh specimens of *R. okamurae* were extracted with acetone/methanol (MeOH) and, after evaporation of the solvent under reduced pressure, the aqueous residue was extracted with diethyl ether (Et_2_O). The resulting extract was subjected to column chromatography (CC) using hexane/Et_2_O mixtures, then Et_2_O and finally CHCl_3_/MeOH mixtures. The obtained fractions were further separated by CC and HPLC to yield the new diterpenoids **1**–**10** (Figure 1).

Rugukadiol A (**1**) possessed the molecular formula C_24_H_36_O_6_, determined by HRESIMS, which indicated seven unsaturation degrees for the molecule. The presence of two acetoxy groups was readily inferred from the singlets in the ^1^H NMR spectrum at δ_H_ 2.09 and 2.03 (Table 1). The spectrum showed another four methyl groups, three linked to double bonds (δ_H_ 1.70, 1.67 and 1.64) and another to a methine (δ_H_ 0.92, d, *J* = 7.0 Hz). The ^13^C NMR spectrum exhibited, besides the four signals due to the acetoxy groups, twenty resonances attributable to a diterpenoid containing two double bonds (δ_C_ 138.3, 132.0, 127.2, 124.2) and four oxygenated carbons (δ_C_ 89.3, 82.2, 82.1, 76.3). Since the acetoxy groups and the double bonds accounted for four unsaturations, the compound must be tricyclic.

**Figure 2:**
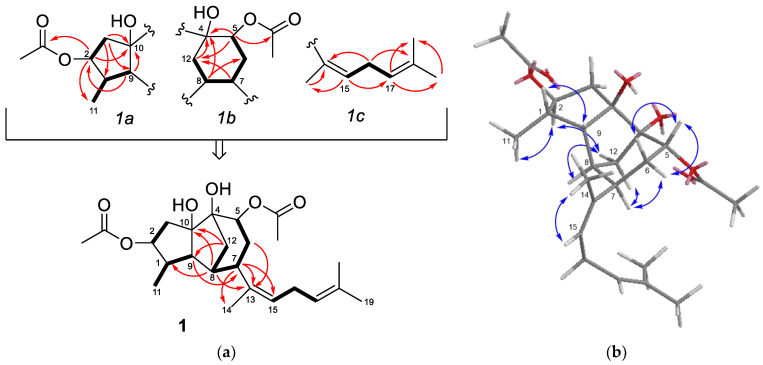
(**a**) Substructures *1a*, *1b*, and *1c* defined for compound **1** and full planar structure; key COSY correlations are shown with bold bonds and key HMBC correlations with arrows; (**b**) key NOESY correlations observed for compound **1**.

The presence of a five-membered ring bearing an acetoxy group, a methyl group, and a tertiary hydroxy group (*1a* in Figure 2a) was supported by the HMBC correlations of the oxymethine proton at δ_H_ 4.66 (H-2) with the carbonyl carbon at δ_C_ 172.9 (-COO-), the methyl carbon at δ_c_ 13.4 (C-11), and the oxygenated carbon at δ_C_ 89.3 (C-10). On the other hand, the presence of a six-membered ring bearing the second acetoxy group and another tertiary hydroxy group (*1b* in Figure 2a), was deduced from the HMBC correlations of the oxymethine proton at δ_H_ 5.23 (H-5) with the acetate carbon at δ_C_ 172.9 (-COO-), the oxygenated carbon at δ_C_ 82.1 (C-4) and the methylene at δ_C_ 39.8 (C-12), which in turn was correlated with a methine proton at δ_H_ 2.88 (H-7); in addition, the COSY couplings connected this methine (H-7) with the oxymethine proton δ_H_ 5.23 (H-5) through a methylene (H-6). In the ^1^HNMR spectrum, the two remaining deshielded protons at δ_H_ 5.18, br t, *J* = 7.4 Hz (H-15) and δ_H_ 5.06, br t, *J* = 7.2 Hz (H-17), were assigned to the protons of two trisubstituted double bonds. These, together with the three allylic methyl groups mentioned above, were accommodated in a regular isoprenoid chain with the two double bonds separated by a methylene (*1c* in Figure 2a).

The three moieties defined for compound **1**: a five-membered ring (*1a*), a six-membered ring (*1b*), and an isoprenoid chain (*1c*), were connected to yield the planar structure of **1** (Figure 2a). In particular, the HMBC correlations of the methine proton H-7 with two olefinic carbons (C-13, C-15) allowed the linking of the side chain to C-7 on the six-membered ring. Finally, C-4 and C-8 on this ring were connected to C-10 and C-9, respectively, on the five-membered ring to yield a tricyclic system containing a methylene bridge (C-12). This proposal was supported, among others, by the HMBC correlations H-12/C-9,C-10, H-8/C-1,C-10 and H-9/C-7. To the best of our knowledge, compound **1** exhibit an unprecedented diterpenoid carbon skeleton.

The relative configuration of **1** was proposed by analysis of the NOESY spectrum (Figure 2b). Thus, the correlations H-2/Me-11, H-2/H-12b, and H-7/H-12a, indicated that H-2, H-7, Me-11, and the methylene bridge were located on the same side of the molecule. The correlation H-1/H-9 supported the *cis*-relationship between these protons on the other side. The NOESY correlations of H-5 with H-6a and H-6b, while H-7 only was correlated with H-6b, was consistent with an equatorial orientation of H-5 and a *trans*-relationship between H-5 and H-7. The *Z* configuration of the double bond at C-13,C-15 was indicated by the correlation Me-14/H-15.

Rugukamural A (**2**) was isolated as an oil whose molecular formula C_24_H_32_O_6_ was established by HRESIMS. The ^13^C NMR spectrum exhibited twenty-four carbon atoms, four of which were due to two acetoxy groups [δ_C_ 172.2 (CH_3_*C*OO−), 172.1 (CH_3_*C*OO−), 21.0 (*C*H_3_COO−) and 20.9 (*C*H_3_COO−)] (Table 2). The remaining twenty carbon signals were attributable to a diterpenoid containing a ketone group (δ_C_ 212.8), an aldehyde group (δ_C_ 201.0), three double bonds (δ_C_ 170.7, 144.5, 139.0, 132.3, 125.9, 113.2) and two methine carbons linked to the acetoxy groups mentioned above (δ_C_ 78.3, 77.8). Taking into account these functional groups and the nine unsaturations calculated from the molecular formula, the compound had to be bicyclic. This datum, together with the presence of the ketone and the aldehyde groups, suggested that compound **2** could be related to the known diterpenoids dilkamural (**11**) and **12**, also isolated from the alga. These compounds feature a bicyclic skeleton known as secospatane, which so far seems exclusive of marine diterpenes of the *Rugulopteryx* species [31,32,33,34,37].

**Figure 3:**
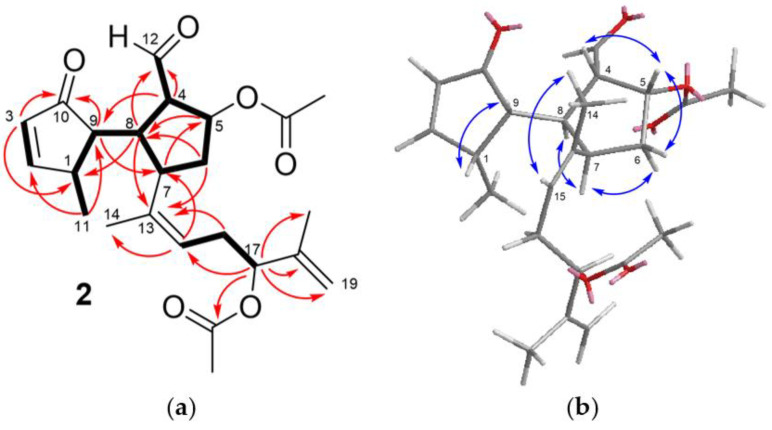
(**a**) Key COSY (bold bond) and HMBC correlations (arrow) observed for compound **2**; (**b**) key NOESY correlations observed for compound **2**.

This proposal was confirmed by analysis of COSY, HSQC, and HMBC spectra (Figure 3a), which indicated the presence of both a conjugated cyclopentenone moiety bearing a methyl substituent and a ciclopentanecarbaldehyde moiety bearing one of the acetoxy groups of the molecule. The linkage of the rings through a single bond was inferred from the COSY coupling between H-8 and H-9 and the HMBC correlations H-4/C-9, H-8/C-1 and H-9/C-7. The remaining carbon atoms of the diterpene and the second acetoxy group were located in an isoprenoid chain linked to C-7, in agreement with the secospatane skeleton of **2**. The HMBC correlations of the oxymethine proton at δ_H_ 5.12 (H-17) with the carbonyl carbon at δ_C_ 172.1, the olefinic methylene at δ_C_ 113.2 (C-19), and an allylic methyl group (δ_C_ 18.7, Me-20), indicated the location of the acetoxy group at C-17 and a terminal double bond at C-18,C19.

The NOESY spectrum of **2** showed the correlations H-4/H-5, H-5/H-6a, H-6b/H-7, H-7/H-8, and H-1/H-9, which indicated that the relative configuration of carbons on the rings was identical to that of dilkamural (**11**) [33], and the correlation Me-14/H-15 defined the *Z* configuration of the trisubstituted double bond at C-13,C-15 (Figure 3b). Based on biogenetic considerations, the stereochemistry of one ring with respect to the other and the absolute configuration were assumed to be identical to that of compounds **11** and **12** [33].

Rugukamural B (**3**) possessed the molecular formula C_22_H_30_O_5_, determined by HRESIMS. The analysis of the NMR spectra (Table 2) indicated that compound **3** displayed the same bicyclic system as compound **2** and that the side chain contained a tertiary alcohol and two double bonds with three olefinic protons in total. The HMBC correlations of the methyl groups at the end of the chain (Me-19 and Me-20) with the oxygenated carbon (δ_C_ 82.6, C-18) and with an olefinic carbon at δ_C_ 138.7 (C-17) was consistent with the location of the tertiary alcohol at C-18 and one of the double bonds at C-16,C-17. The sequence of COSY couplings among the three olefinic protons indicated that the two double bonds were conjugated. The 13*Z*,17*E* configuration was defined from the NOESY correlation between H-15 and Me-14, and the coupling constant of 15.5 Hz between H-16 and H-17, respectively.

The NMR data of compound **4** (Table 2) were closely related to those of dilkamural (**11**) [36], except for the absence of the signals of the acetyl groups and the significant shielding of protons H-2 (δ_H_ 4.11 vs. δ_H_ 5.02 in **11**) and H-5 (δ_H_ 4.76 vs. δ_H_ 5.63 in **11**). These data, together with the molecular formula C_20_H_30_O_4_ established by HRMS and the correlations observed in the NOESY spectrum, confirmed that compound **4** was the deacetyl derivative of dilkamural.

**Figure 4:**
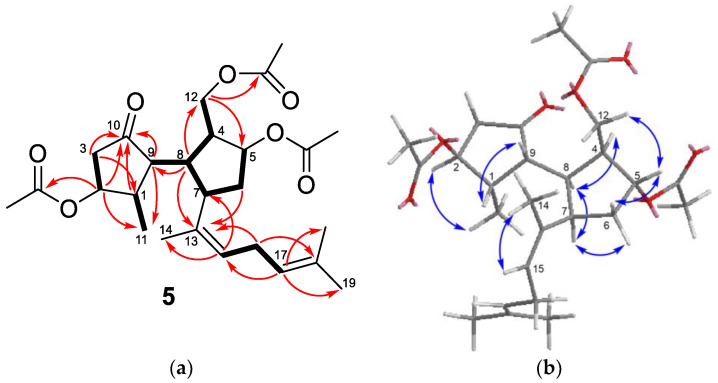
(**a**) Key COSY (bold bond) and HMBC (arrow) correlations observed for compound **5**; (**b**) key NOESY correlations observed for compound **5**.

Ruguloptone A (**5**) possessed the molecular formula C_26_H_38_O_7_, determined by HRESIMS. The presence of three acetoxy groups in the molecule was readily defined from the ^1^H NMR signals at δ_H_ 2.048 (s, 3H), 2.045 (s, 3H), and 2.04 (s, 3H) (Table 3). The spectrum showed the signals of another four methyl groups, three of them linked to double bonds (δ_H_ 1.73, 1.68, 1.63) and the remaining one linked to a methine (δ_H_ 0.92, d, *J* = 7.3 Hz). Differing from rugukamurals A–C (**2**–**4**), the ^1^HNMR spectrum of compound **5** did not show any aldehyde proton signal. In the ^13^C NMR spectrum, besides the six signals due to the acetoxy groups, there were twenty signals attributable to a diterpene containing a ketone carbonyl, two double bonds and three carbons linked to the acetoxy groups mentioned above (one methylene at δ_C_ 64.0 and two methines at δ_C_ 79.8 and 76.8). The HMBC correlations of the ketone carbonyl (C-10) with one oxymethine proton (δ_H_ 5.01, H-2) and with the methine geminal to methyl (δ_H_ 2.77, H-1) were consistent with the presence of a cyclopentanone ring bearing one methyl and one of the acetoxy groups (Figure 4a). The remaining two acetoxy groups were accommodated on another five-membered ring. Key correlations were the sequence of couplings in the COSY spectrum from the oxymethylene protons at δ_H_ 4.09 and 4.03 (H-12a and H-12b) through three methines (H-4, H-8, H-7) and one methylene (H-6) to reach the oxymethine proton at δ_H_ 5.23 (H-5), and the HMBC correlation of the oxymethylene protons (H-12) with the oxymethine carbon (δ_C_ 79.8, C-5). The two double bonds and the three remaining methyl groups were located on a regular isoprenoid chain identical to that of compound **4**. The HMBC correlations of H-8 with C-9 and C-13 confirmed the linkage of the two rings of **5** and the position of the side chain at C-7, in agreement with a secospatane framework. The relative configuration of compound **5** was defined from the correlations observed in the NOESY spectrum (Figure 4b). The correlations H-1/H-9 and H-2/Me-11 indicated that H-1 and H-2 were *trans*, while H-1 ad H-9 were *cis*. For the other ring, the correlations H-5/H-12b, H-5/H-6a, H-4/H-8, H-8/H-7, and H-7/H-6b were consistent with the location of H-5 and H-12 in the same side of the ring, and of H-4, H-7, and H-8 on the other side. The correlation Me-14/H-15 indicated the *Z* configuration of the double bond at C-13,C-15.

The NMR spectra of ruguloptone B (**6**) (Table 3) were closely similar to those of compound **5**, except for the presence of only two acetoxy groups and the shielding of H-2 (δ_H_ 4.10 vs. 5.01 in **5**), which indicated that compound **6** was the 2-deacetyl derivative of **5**.

Ruguloptone C (**7**) possessed the molecular formula C_24_H_35_O_5_, determined by HRESIMS. Differing from compound **5**, the NMR spectra of **7** (Table 3) showed the signals of two secondary acetoxy groups and lacked the signals of the oxymethylene at C-12, showing, in turn, those of an exomethylene at δ_C_ 113.4 (C-12)/δ_H_ 5.27 (H-12a) and 5.03 (H-12b). Therefore, **7** was the analogue of **5** containing a double bond at C-4,C-12.

The molecular formula of ruguloptone D (**8**), C_22_H_32_O_3_, and the NMR spectra (Table 4) suggested that it was another secospatane diterpenoid related to those described above, containing a ketone group, a primary acetoxy group and three double bonds. A diene-containing side chain identical to that of compounds **4**–**7** was identified in **8**. The third double bond of the molecule was located at C-4,C-5 and the acetoxy group at C-12 from the HMBC correlations of the oxymethylene protons [δ_H_ 4.74 (H-12a) and 4.67 (H-12b)] with the methine carbon C-8 and with the olefinic carbon at δ_C_ 130.5 (C-5), which was also correlated with the methine proton H-7, geminal to the side chain. The remaining carbon atoms and the carbonyl group were located in a ciclopentanone ring. The correlations in the NOESY spectrum indicated that **8** possessed at C-1, C-7, C-8, and C-9, the same relative configuration as compounds **2**–**7**.

Ruguloptone E (**9**) exhibited NMR spectra (Table 4) closely similar to those of compound **8**, except for the absence of the signals due to the acetoxy group. The spectra showed, in turn, the signals of a methyl linked to a double bond [δ_C_ 16.6 (C-12)/δ_H_ 1.68 (H-12)] (Table 4), which were consistent with the presence of a methylcyclopentene moiety.

The last compound of this series was ruguloptone F (**10**), which possessed the molecular formula C_22_H_32_O_4_. The NMR spectra (Table 4) were related to those of the known compound **12**, except for the absence of the aldehyde signals, showing in turn those of an oxymethylene at δ_C_ 62.0 (C-12)/δ_H_ 3.46 (H-12a) and 3.42 (H-12b). These, and the remaining NMR data, supported that compound **10** was the primary alcohol derived from reduction of the aldehyde of **12**. The NOESY correlations indicated that the relative configuration of **10** was identical to that of **12**. A compound exhibiting the same planar structure and relative configuration at C-1, C-7, C-8, and C-9 as **10** was reported from *R. marginata* (formerly *Dilophus marginatus*), while the relative configuration at C-4 and C-5 was not defined [37]. The NMR data of **10** did not match those reported for the compound from of *R. marginata,* indicating they must be isomers.

### 2.2. Anti-Inflammatory Activity

The new compounds **1**, **4**, **5**, **6**, **7**, **10**, and the known compounds **11** and **12** were tested for their anti-inflammatory activity, in particular as inhibitors of nitric oxide (NO) production and of classical pro-inflammatory cytokines expression.

Anti-inflammatory assays were performed on immune cells Bv.2 cells (microglia) and RAW 264.7 cells (macrophages), which are key mediators in inflammatory processes. The stimulation of these cells by bacterial products such as lipopolysaccharide (LPS) promotes the synthesis and release of NO and pro-inflammatory cytokines, which are intermediaries involved in the inflammatory onset [38,39]. Since high concentrations of NO are essential for inflammation and related processes, targeting the inducible nitric oxide synthase (iNOS), which is the enzyme responsible for NO synthesis, has been proposed as an anti-inflammatory therapeutic strategy [40].

First, the cytotoxicity of different concentrations of the compounds towards Bv.2 and RAW 264.7 cells was checked. Compounds **1**, **4**, **5**, **6**, and **10** at concentrations equal or lower than 10 μM were not cytotoxic for Bv.2 and RAW 264.7 cells, while compound **4** showed cytotoxic effects on macrophages and compound **7** on both cell lines (Figures S21 and S22). Compounds **2** and **8** were not tested because of paucity of material and compound **9** by solubility issues. Compound **3** was expected to be cytotoxic as it was the close analogue **12** (see below) and was discarded for the assays.

**Figure 5:**
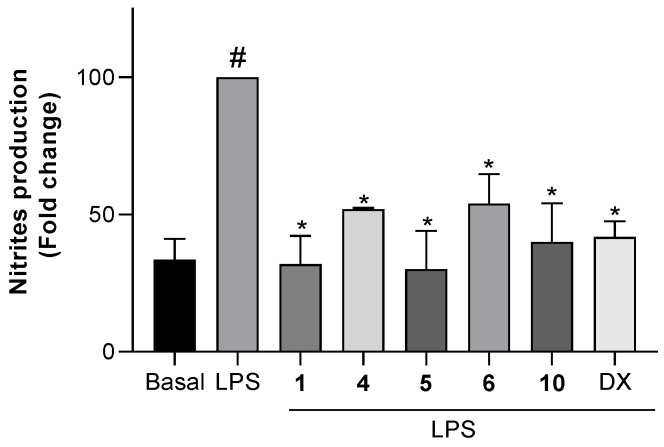
Effects of compounds **1**, **4**, **5**, **6**, or **10** on NO release in microglial cells. Bv.2 microglial cells were pretreated for 3 h with the compound at 10 μM, followed by stimulation with 200 ng/mL LPS for 24 h. Nitrite accumulation in the culture media was measured using the Griess method. Results are expressed as a fold change relative to the LPS condition and are mean ± SD (n ≥ 3 independent experiments performed in duplicate). Significant differences were determined by two-way ANOVA followed by Bonferroni *t*-test; * *p* ≤ 0.05 vs. LPS; ^#^ *p* ≤ 0.05 vs. Basal.

**Figure 6:**
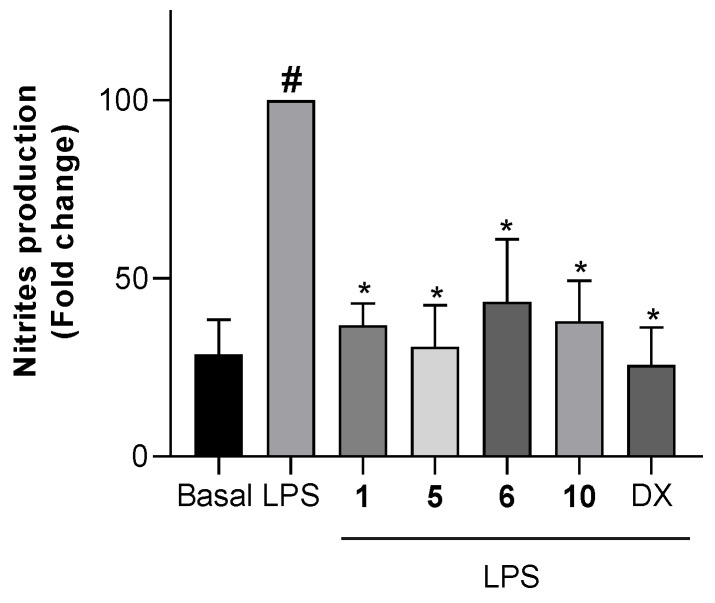
Effects of compounds **1**, **5**, **6**, or **10** on NO release in macrophage cells. RAW 264.7 macrophage cells were pretreated for 3 h with the selected compound at 10 μM, followed by stimulation with 200 ng/mL LPS for 24 h. Nitrite accumulation in the culture media was measured using the Griess method. Results are expressed as fold change relative to the LPS condition and are mean ± SD (n ≥ 3 independent experiments performed in duplicate). Significant differences were determined by two-way ANOVA followed by Bonferroni *t*-test; * *p* ≤ 0.05 vs. LPS; ^#^ *p* ≤ 0.05 vs. Basal.

To test the effects of the non-cytotoxic compounds **1**, **4**, **5**, **6**, and **10** on the production of NO, cells were pretreated with the compounds, then stimulated with LPS and, finally, the concentration of nitrite, which is the major metabolite formed from NO, was measured. The level of nitrites in both microglial and macrophage cells treated with the compounds but not further stimulated, did not change with respect to untreated cells. The levels of nitrites in Bv.2 and RAW 264.7 cells treated with the compounds and further LPS-stimulated are shown in Figure 5 and Figure 6, respectively.

As shown in Figure 5, upon treatment of control Bv.2 cells (Basal) with LPS, the level of nitrites was significantly increased (column LPS). However, in cells pretreated for 3 h with compounds **1**, **4**, **5**, **6**, or **10**, the LPS-stimulated production of nitrites was significantly inhibited. Compounds **1**, **5**, and **10** were highly active, causing, at 10 μM, 68.1%, 70.0%, and 60.0% inhibition of nitrite production, respectively, with respect to stimulated and untreated cells. The inhibitory effects of **1** and **5** were higher than those caused by the reference compound dexamethasone at 2.5 μM. Compounds **4** and **6** were less active, causing 48.1% and 46.0% inhibition, respectively.

On the other hand, dilkamural (**11**) and compound **12** exhibited significant cytotoxicity at 10 μM (**11** cytotoxic even at 1 μM) against Bv.2 cells (Figure S21), and were tested at the maximum concentration of 0.5 μM. While **11** did not show any significant effect on the NO production, compound **12** caused 30% of inhibition.

Assays with RAW 264.7 cells showed a similar outcome (Figure 6). Treatment of cells with LPS significantly increased the level of nitrites. The pretreatment with compounds **1**, **5**, and **10** at 10 μM inhibited the LPS-stimulated production of nitrites by 63.2%, 69.2%, and 64.9%, respectively. These effects were slightly lower than those caused by dexamethasone at 2.5 μM. Compound **6** was again the less active, causing 56.6% inhibition of nitrite production.

These results show the anti-inflammatory potential of the new diterpenoids rugukadiol A (**1**), ruguloptone A (**5**), and ruguloptone F (**10**), which are capable of counteracting almost completely the effects of the LPS stimulation on cells, maintaining NO concentrations close to basal levels (non-stimulated cells). Moreover, among the tested secospatanes, it seems that the presence of a primary hydroxy or acetoxy group at C-12 correlates with the anti-inflammatory activity.

In order to obtain further data on the anti-inflammatory effects, compounds **1** and **5**, which showed a potent inhibitory activity of NO secretion, were selected to analyze the inhibition of *Nos2* and pro-inflammatory cytokines expression.

**Figure 7:**
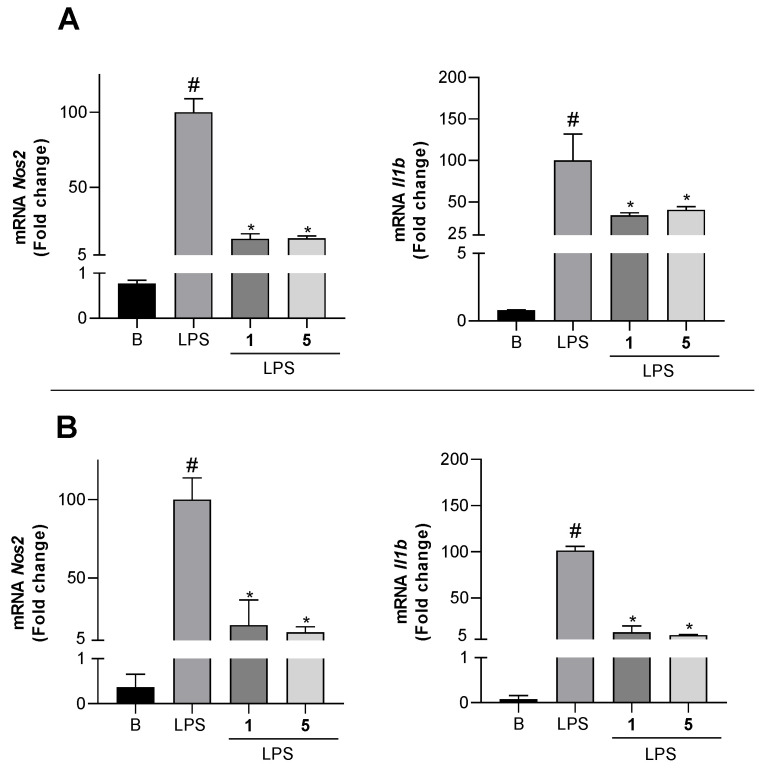
Inhibitory effects of compounds **1** and **5** on mRNA pro-inflammatory cytokines expression. (**A**) *Nos2, Il1b*, and *Actin-b* mRNA levels in Bv.2 microglial cells were determined by qRT-PCR. (**B**) *Nos2*, *Il1b*, and *Actin-b* mRNA levels in RAW 264.7 macrophage cells were determined by qRT-PCR. Results are expressed as fold change relative to the LPS condition and are mean ± SD (n ≥ 3 independent experiments performed in duplicate). Significant differences were determined by two-way ANOVA followed by Bonferroni *t*-test; * *p* ≤ 0.05 vs. LPS; ^#^ *p* ≤ 0.05 vs. Basal.

As shown in Figure 7A, significant increases in the mRNA expression of *Nos2* and *Il1b* in Bv.2 cells were detected after LPS stimulus (column LPS). However, the pretreatment of cells with compounds **1** and **5** at 10 μM decreased *Nos2* mRNA levels by 82.6% and 83.9%, respectively, as well as *Il1b* mRNA levels by 66.2% and 59.5%, respectively. Similarly, the LPS-stimulated expression of *Nos2* and *Il1b* in RAW 264.7 cells was significantly inhibited by compounds **1** and **5** (Figure 7B). The inhibition of mRNA *Nos2* caused by both compounds in macrophages was similar to that observed in Bv.2 cells (80–85% inhibition of mRNA levels), but the effects were much stronger on *Il1b,* which was inhibited by compounds **1** and **5** up to 87.1% and 90.4%, respectively. On the other hand, no inhibition of *Tnfa* expression was detected in any of the cells. This differential response of cytokines expression to the anti-inflammatory effects of compounds **1** and **5** could be involved in the complex signaling network that modulate the inflammatory response.

The overproduction of NO that causes inflammatory tissue damage may be suppressed through inhibition of the L-arginine/nitric oxide pathway by different mechanisms, which include the inhibition of iNOS or the competition with arginine [41]. In this study, the decreased levels of NO production in Bv.2 and RAW 264.7 cells treated with compounds **1** and **5** were associated with the inhibition of *Nos2* expression. Some marine diterpenoids have been shown to exert anti-inflammatory effects by inhibiting the NFκB signaling pathway at different levels [42]. NFκB is a transcription factor that regulates the transcription of pro-inflammatory cytokine genes [43]. In this regard, the effects observed on *Il1b* expression while mRNA levels of *Tnfa* were not affected suggested that the anti-inflammatory activity of compounds **1** and **5** could be related to the modulation of inflammasome complex activation [44,45].

The pro-inflammatory response of immune cells (macrophages and microglia cells) to LPS, known as M1 or classical activation, functions predominantly in situations of tissue damage [46]. This complex signaling network is working by the generation of different inflammatory mediators such as tumor necrosis factor α (TNFα), interleukins 1β and 6 (IL1β, IL6) and NO [47], that contribute to enhance the pro-inflammatory response. However, the immunomodulatory properties that compounds **1** and **5** exert on specific signaling pathways invite to analyze the mechanisms involved in the induced anti-inflammatory response.

Most of the marine diterpenoids with anti-inflammatory properties have been obtained from octocorals, and belong to the eunicellane, briarane, cembrane, serrulatane, amphilectane, lobane, verticillane and pseudopterane skeletal classes [42]. This study on algal diterpenoids is the first account of anti-inflammatory activity within the secospatane class of metabolites, and also adds a new structural class represented by compound **1**, thus extending the range of biological sources and structural variety of marine diterpenoids with anti-inflammatory potential.

From the point of view of marine organisms as sources of bioactive compounds, macroalgae may be advantageous over other marine macroorganisms, because of the possibility of obtaining biomass through sustainable wild-harvest or culture [48,49]. Currently, the alga *R. okamurae* that invades the Strait of Gibraltar produces huge amounts of biomass that causes highly detrimental effects in the region, both environmental and economic [26,27]. This has led to the search of strategies aimed to control the spread of the alga or to diminish its negative effects. In this line, recent reports have highlighted the opportunities offered by invasive macroalgae to obtain valuable products [50,51,52,53]. Our results have shown that *R. okamurae* contains an array of compounds, some which could be of interest for pharmacological purposes in the anti-inflammatory area. It is also worth noting that *R. okamurae* from the Strait of Gibraltar contains high concentrations of the diterpene dilkamural (**11**) and of its elimination product **12**. Although these compounds may be disregarded for further anti-inflammatory studies, they could exhibit suitable properties in other therapeutic areas. In addition, **11** and **12** may be highly valuable starting materials to synthesize more active but less abundant compounds and other analogues. Thus, in this study we performed a reduction reaction of **12** with NaBH_4_ that allowed obtaining additional amounts of compound **10** for biological testing. Currently, further biological activities of **11** and **12**, as well as other chemical transformations of these compounds, are under study.

## 3. Materials and Methods

### 3.1. General Experimental Procedures

Optical rotations were measured on a Jasco P-2000 polarimeter (Jasco, Easton, MD, USA). IR spectra were recorded on a Perkin-Elmer FT-IR Spectrum Two spectrometer (Perkin Elmer, Boston, MA, USA). ^1^H and ^13^C NMR spectra were recorded on an Agilent 500 (Agilent Technologies, Santa Clara, CA, USA) or on a Bruker 500 spectrometer (Bruker, Billerica, MA, USA) using CD_3_OD as solvent. Chemical shifts were referenced using the solvent signals at δ_H_ 3.30 and δ_C_ 49.0. COSY, HSQC, HMBC, and NOESY experiments were performed using standard Agilent or Bruker pulse sequences. High resolution mass spectra (HRESIMS) were obtained on a Waters XEVO G2-S Mass spectrometer (Waters, Milford, MA, USA). Column chromatography was carried out on Merck Silica gel 60 (70–230 mesh) (Merck, Darmstadt, Germany). SPE separations were performed on Supelco DSC18 cartridges (500 mg/3 mL or 1 g/6 mL) (Supelco, Bellefonte, PA, USA). HPLC separations were performed on a LaChrom-Hitachi apparatus (Merck, Darmstadt, Germany) using a differential refractometer RI-71. Luna Si (2) (250 × 4.6 mm, 5 μm) (Phenomenex, Torrance, CA, USA) and Luna Si (2) (250 × 10 mm, 5 μm) (Phenomenex, Torrance, CA, USA) columns were used for separations in normal phase. All solvents were of HPLC grade.

### 3.2. Algae Collection

Specimens of *R. okamurae* (E.Y. Dawson) I. K. Hwang, W. J. Lee and H. S. Kim (Class Phaeophyceae, Order Dictyotales, Family Dictyotaceae) were collected at Punta Carnero (Cádiz, Spain, 36°04′38.6″ N; 5°25′31.1″ W) and transported to the laboratory in a thermal refrigerator. Algae were washed with fresh water to remove epiphytes and organic and inorganic debris and immediately extracted. A voucher specimen (RO-1019) is deposited at the Marine Natural Products Laboratory, Faculty of Marine and Environmental Sciences, University of Cadiz, Spain.

### 3.3. Extraction and Isolation 

Fresh samples of *R. okamurae* (500 g) were extracted with acetone/MeOH (1:1, *v*/*v*, 1.5 L) at room temperature. The solvent was evaporated under reduced pressure and the aqueous residue was extracted with Et_2_O (4 × 100 mL). The Et_2_O layers were combined, dried over MgSO_4_, and evaporated under reduced pressure to yield 8.2 g of extract. The Et_2_O extract was subjected to silica gel column chromatography (28 × 5.5 cm) using as eluents hexanes/Et_2_O (9:1, *v*/*v*, 0.8 L), hexanes/Et_2_O (8:2, *v*/*v*, 1.0 L), hexanes/Et_2_O (7:3, *v*/*v*, 2.0 L), hexanes/Et_2_O (1:1, *v*/*v*, 1.5 L), hexanes/Et_2_O (3:7, *v*/*v*, 1.0 L), Et_2_O (1.5 L), CHCl_3_/MeOH (8:2, *v*/*v*, 1.0 L) and finally MeOH (0.7 L). The fraction that eluted with hexanes/Et_2_O (9:1, *v*/*v*) was separated over a silica gel column using n-hexane/Et_2_O mixtures (99:1 to 9:1 *v*/*v*) and Et_2_O. The subfractions that in their respective ^1^H NMR spectra showed signals attributable to diterpenes were purified by HPLC (n-hexane/EtOAc, 99:1, *v*/*v*) yielding compound **9**. The fraction that eluted with hexanes/Et_2_O (7:3, *v*/*v*) was separated by silica gel column chromatography using n-hexane/Et_2_O mixtures (95:5 to 6:4, *v*/*v*) and AcOEt. The subfractions that in their ^1^H NMR spectra showed signals attributable to terpenoids were subjected to repeated purifications by normal-phase HPLC (n-hexane/EtOAc, 95:5 and 85:15, *v*/*v*), yielding compounds **8**, **7**, and **12**. The fraction that eluted with hexanes/Et_2_O (1:1, *v*/*v*) was suspended in MeOH/H_2_O (9:1, *v*/*v*, 6 mL) and transferred onto six SPE-C18 cartridges preconditioned with MeOH/H_2_O (9:1, *v*/*v*, 1 mL each cartridge). Each cartridge was eluted with 10 mL of MeOH/H_2_O (9:1, *v*/*v*). The resulting solution was evaporated under reduced pressure yielding a mixture (2.3 g) that was subjected to HPLC separation (n-hexane/EtOAc (7:3, *v*/*v*) to yield compounds **5**, **11**, and additional amounts of **12**. The fraction that eluted with hexanes/Et_2_O (3:7, *v*/*v*) was subjected to silica gel column chromatography using n-hexane/Et_2_O mixtures (8:2 to 1:1, *v*/*v*) and MeOH. The subfractions that in their ^1^H NMR spectra showed signals attributable to terpenoids were further purified by HPLC (n-hexane/EtOAc, 8:2, 7:3 and 6:4, *v*/*v*) yielding compounds **2** and **10** and further amounts of **11** and **12**. The fraction that eluted with Et_2_O was suspended in MeOH/H_2_O (9:1, *v*/*v*, 2 mL) and transferred onto two SPE-C18 cartridges preconditioned with MeOH/H_2_O (9:1, *v*/*v*, 1 mL). Each cartridge was eluted with 10 mL of MeOH/H_2_O (9:1, *v*/*v*). The resulting solution was evaporated under reduced pressure yielding a mixture (457.5 mg) that was separated over a silica gel column using n-hexane/Et_2_O mixtures (75:25 to 1:1, *v*/*v*) and AcOEt. The subfractions that in their respective ^1^H NMR spectra showed signals attributable to terpenoids were purified by HPLC (n-hexane/EtOAc, 7:3 and 6:4, *v*/*v*) to yield compounds **1**, **3**, and **6**. The fraction that eluted with CHCl_3_/MeOH was separated over a silica gel column using n-hexane/Et_2_O mixtures (6:4 to 4:6, *v*/*v*), AcOEt and MeOH. Selected fractions were purified by HPLC (n-hexane/EtOAc, 7:3 and 6:4, *v*/*v*) yielding compound **4** and further amounts of **1**. The total amounts obtained of each compound were **1** (54.5 mg), **2** (3.5 mg), **3** (10.0 mg), **4** (12.7 mg), **5** (80.0 mg), **6** (20.1 mg), **7** (7.1 mg), **8** (4.7 mg), **9** (14.5 mg), **10** (2.0 mg), **11** (964.8 mg), and **12** (781.7 mg).

### 3.4. Characterization of Compounds

Rugukadiol A (**1**): colorless oil; ${\left[\alpha \right]}_{D}^{25}$ +30.5 (*c* 0.09, MeOH); IR (film) υ_max_ 3431, 2964, 1736, 1240 cm^−1^; ^1^H NMR (CD_3_OD, 500 MHz) Table 1; ^13^C NMR (CD_3_OD, 125 MHz) Table 1; HRESIMS *m*/*z* 443.2421 [M + Na]^+^ (calcd. for C_24_H_36_O_6_Na 443.2410).

Rugukamural A (**2**): colorless oil; ${\left[\alpha \right]}_{D}^{25}$ +58.4 (*c* 0.11, MeOH); IR (film) υ_max_ 3357, 2931, 1736, 1248 cm^−1^; ^1^H NMR (CD_3_OD, 500 MHz) Table 2; ^13^C NMR (CD_3_OD, 125 MHz) Table 1; HRESIMS *m*/*z* 439.2102 [M + Na]^+^ (calcd. for C_24_H_32_O_6_Na, 439.2097).

Rugukamural B (**3**): colorless oil; ${\left[\alpha \right]}_{D}^{25}$ +20.7 (*c* 0.08, MeOH); IR (film) υ_max_ 3357, 2931, 1732, 1240 cm^−1^; ^1^H NMR (CD_3_OD, 500 MHz) Table 2; ^13^C NMR (CD_3_OD, 125 MHz) Table 1; HRESIMS *m*/*z* 397.1996 [M + Na]^+^ (calcd. for C_22_H_30_O_5_Na 397.1991).

Rugukamural C (**4**): colorless oil; ${\left[\alpha \right]}_{D}^{25}$ +12.9 (*c* 0.13, MeOH); IR (film) υ_max_ 3357, 2931, 1732, 1375 cm^−1^; ^1^H NMR (CD_3_OD, 500 MHz) Table 2; ^13^C NMR (CD_3_OD, 125 MHz) Table 1; HRESIMS *m*/*z* 357.2053 [M + Na]^+^ (calcd. for C_20_H_30_O_4_Na 357.2042). 

Ruguloptone A (**5**): colorless oil; ${\left[\alpha \right]}_{D}^{25}$ +9.6 (*c* 0.12, MeOH); IR (film) υ_max_ 2967, 1735, 1237 cm^−1^; ^1^H NMR (CD_3_OD, 500 MHz) Table 3; ^13^C NMR (CD_3_OD, 125 MHz) Table 3; HRESIMS *m*/*z* 485.2527 [M + Na]^+^ (calcd. for C_26_H_38_O_7_Na 485.2515).

Ruguloptone B (**6**): colorless oil; ${\left[\alpha \right]}_{D}^{25}$ +13.7 (*c* 0.09, MeOH); IR (film) υ_max_ 3426, 2966, 1733, 1241 cm^−1^; ^1^H NMR (CD_3_OD, 500 MHz) Table 3; ^13^C NMR (CD_3_OD, 125 MHz) Table 1; HRESIMS *m*/*z* 443.2432 [M + Na]^+^ (calcd. for C_24_H_36_O_6_Na 443.2410).

Ruguloptone C (**7**): colorless oil; ${\left[\alpha \right]}_{D}^{25}$ +24.4 (*c* 0.19, MeOH); IR (film) υ_max_ 2965, 1734, 1234, cm^−1^; ^1^H NMR (CD_3_OD, 500 MHz) Table 3; ^13^C NMR (CD_3_OD, 125 MHz) Table 3; HRESIMS *m*/*z* 425.2304 [M + Na]^+^ (calcd. for C_24_H_34_O_5_Na 425.2304).

Ruguloptone D (**8**): colorless oil; ${\left[\alpha \right]}_{D}^{25}$ +38.6 (*c* 0.14, MeOH); IR (film) υ_max_ 2962, 1260 cm^−1^; ^1^H NMR (CD_3_OD, 500 MHz) Table 4; ^13^C NMR (CD_3_OD, 125 MHz) Table 4; HRESIMS *m*/*z* 367.2258 [M + Na]^+^ (calcd. for C_22_H_32_O_3_Na 367.2249).

Ruguloptone E (**9**): colorless oil; ${\left[\alpha \right]}_{D}^{25}$ +54.9 (*c* 0.08, MeOH); IR (film) υ_max_ 2962, 1742 cm^−1^; ^1^H NMR (CD_3_OD, 500 MHz) Table 4; ^13^C NMR (CD_3_OD, 125 MHz) Table 4; HRESIMS *m*/*z* 309.2195 [M + Na]^+^ (calcd. for C_20_H_30_ONa 309.2194).

Ruguloptone F (**10**): colorless oil; ${\left[\alpha \right]}_{D}^{25}$ −70.8 (*c* 0.1, MeOH); IR (film) υ_max_ 3433, 2967, 1733, 1696, 1242 cm^−1^; ^1^H NMR (CD_3_OD, 500 MHz) Table 4; ^13^C NMR (CD_3_OD, 125 MHz) Table 4; HRESIMS *m*/*z* 383.2200 [M + Na]^+^ (calcd. for C_22_H_32_O_4_Na 383.2198).

### 3.5. Cell Culture

Mouse microglia Bv.2 cell line was supplied by Dr. M. L. Nieto (IBGM, Spain). Mouse macrophages RAW 264.7 cell line was supplied by Dr. A. M. Valverde (IIBm “Alberto Sols” UAM-CSIC-Madrid, Spain). An amount of 1.5 × 10^5^ cells/well were seeded in a 6-multiwell plate (Sarstedt, Germany). The culture conditions were 37 °C in a humidified atmosphere with 5% CO_2_ in RPMI supplemented with 10% (*v*/*v*) heat-inactivated fetal bovine serum (FBS), 1% (*v*/*v*) penicillin/streptomycin (Sigma), and 2 mM L-glutamine (Gibco, Carlsbad, CA, USA). All experimental cell approaches were performed in complete medium without FBS.

### 3.6. Analysis of the Cellular Viability by Crystal Violeta Staining

Cells were cultured in 24-well plates and grown up to 70% confluence. The cells were treated with solutions of the diterpenes to reach final concentrations of 0.1, 1.0, 10.0, 25.0, and 50.0 µM, and incubated in serum-free medium. After 24 h, the medium was discarded and cells were fixed by adding 0.5 mL of glutaraldehyde 1% (*v*/*v*) for 30 min. Then, the plates were rinsed with phosphate buffer saline (PBS) and the remaining viable adherent cells were stained with crystal violet 0.1% (*w*/*v*) for 30 min. After rinsing plates with water and drying for 24 h, 0.5 mL of acetic acid 10% (*v*/*v*) were added. The absorbance of each plate was read spectrophotometrically at 590 nm in a microplate reader (Versamax Tunable Microplate reader, Molecular Devices, Sunnyvale, CA, USA).

### 3.7. Analysis of Nitrites (NO_2_^−^)

Cells were cultured in 6-well plates and grown up to 70% confluence. The cells were pre-treated for 3 h with the diterpenes at 10 µM in serum-free medium and then stimulated with lipopolysaccharide (LPS, 200 ng/mL) for another 24 h. Dexamethasone (Dx) was used as positive reference compound at 2.5 µM. After cell treatments, levels of NO_2_ were measured by using the Griess method [54]. Briefly, cell cultured medium was treated with an acid solution containing 1% sulphanilamide and 0.1% N-(1-naphthyl) ethylenediamine (NEDA) and read spectrophotometrically at 548 nm in a microplate reader.

### 3.8. Quantitative Real-Time PCR (qPCR) Analysis

Total RNA was extracted with TRIzol^®^ reagent (Invitrogen, Madrid, Spain) and reverse-transcribed using the iScript gDNA Clear cDNA Synthesis Kit from BioRad (Madrid, Spain). qPCR was performed with the iTaq Universal Probes Supermix from BioRad (Madrid, Spain) in a CFX Connect Real-Time System from BioRad (Madrid, Spain). Analysis of relative gene expression data were performed using the 2^−ΔΔCT^ method. Primer–probe sets for mouse *Nos2*, *Il1b*, *Tnfa*, and actin were purchased as predesigned TaqMan gene expression assays (Applied Biosystems, Foster City, CA, USA).

### 3.9. Statistical Analysis

Data are presented as mean ± standard deviation (SD), and were compared by using the Bonferroni ANOVA test. All statistical analyses were performed using GraphPad Prism 8.0 software (GraphPad Software Inc., San Diego, CA, USA) with 2-sided tests. Differences were considered statistically significant at *p* ≤ 0.05.

## 4. Conclusions

The brown alga *R. okamurae* contains a new compound, rugukadiol A (**1**), which exhibits an unprecedented diterpenoid skeleton and displays significant anti-inflammatory activity in immune cells, as inhibitor of the production of NO and of the expression of *Nos2* and the cytokine *Il1b*. Nine new diterpenoids of the secospatane class (**2**–**10**) have also been isolated from this alga. Among them, ruguloptones A (**5**) and F (**10**), exhibiting a primary oxygenated function at C-12, possess significant anti-inflammatory activity. The effects caused by **1** and **5** on specific signaling pathways of inflammation suggest that these compounds and analogues deserve to be further explored in advanced biological assays. In this regard, the abundant biomass of *R. okamurae* that accumulates on the coasts of the Strait of Gibraltar could be a resource for providing these bioactive compounds.

## Figures and Tables

**Table 1:** NMR data of rugukadiol A (**1**) in CD_3_OD ^a,b^.

Position	δ_C_, Type	δ_H_, m (*J* in Hz)	Position	δ_C_, Type	δ_H_, m (*J* in Hz)
1	42.7, CH	2.53, m	11	13.4, CH_3_	0.92, d (7.0)
2	82.2, CH	4.66, ddd (8.6, 8.0, 7.5)	12	39.8, CH_2_	1.93, dd (12.1, 2.0) 1.49, ddd (12.1, 5.8, 1.9)
3	43.6, CH_2_	2.91, dd (14.5, 7.5) 1.57, dd (14.5, 8.0)	13	138.3, C	
4	82.1, C		14	22.4, CH_3_	1.70, d (1.2)
5	76.3, CH	5.23, ddd (4.0, 1.9, 1.9)	15	127.2, CH	5.18, br t (7.4)
6	30.6, CH_2_	2.37, ddd (14.8, 13.4, 4.0) 1.58, m	16	27.7, CH_2_	2.68, m
7	40.1, CH	2.88, dd (13.4, 4.8)	17	124.2, CH	5.06, br t (7.2)
8	38.3, CH	1.97, br d (5.5)	18	132.0, C	
9	53.1, CH	2.41, br d (8.5)	19	25.9, CH_3_	1.67, d (1.1)
10	89.3, C		20	17.9, CH_3_	1.64, br s
CH_3_*CO*O (2)	172.9, C				
*CH_3_*COO (2)	21.0, CH_3_	2.03, s			
CH_3_*CO*O (5)	172.8, C				
*CH_3_*COO (5)	21.5, CH_3_	2.09, s			

^a 1^H at 500 MHz, ^13^C at 125 MHz; ^b^ assignments aided by COSY, HSQC, HMBC, and NOESY experiments.

**Table 2:** NMR data of rugukamurals A–C (**2**–**4**) in CD_3_OD ^a,b^.

Position	2		3		4	
	δ_C_, Type	δ_H_, m (*J* in Hz)	δ_C_, Type	δ_H_, m (*J* in Hz)	δ_C_, Type	δ_H_, m (*J* in Hz)
1	41.5, CH	3.07, m	41.3, CH	3.06, m	43.6, CH	2.51, m
2	170.7, CH	7.73, dd (5.8, 3.0)	170.7, CH	7.68, ddd (5.8, 3.1, 0.6)	73.6, CH	4.11, d (5.8)
3	132.3, CH	5.98, dd (5.8, 1.6)	132.2, CH	5.96, dd (5.8, 1.5)	45.2, CH_2_	2.42, dd (19.0, 5.7) 2.06, br d (19.0)
4	58.6, CH	3.76, ddd (10.8, 6.8, 1.8)	58.3, CH	3.78, ddd (11.2, 7.3, 1.8)	61.6, CH	3.18, ddd (9.8, 6.6, 2.6)
5	77.8, CH	5.67, ddd (6.8, 6.8, 3.5)	77.8, CH	5.69, ddd (7.3, 6.7, 4.1)	75.9, CH	4.76, ddd (6.2, 6.2, 3.5)
6	37.8, CH_2_	2.25, ddd (14.6, 6.3, 5.6) 1.93, m	37.2, CH	2.27, ddd (14.7, 6.7, 4.7) 1.95, m	41.0, CH_2_	2.02, m 1.79, m
7	42.0, CH	3.63, ddd (8.8, 8.6, 5.6)	42.4, CH	3.84, ddd (8.7, 8.7, 4.7)	41.6, CH	3.64, m
8	39.5, CH	3.01, ddd (10.8, 9.1, 8.8)	40.0, CH	3.06, m	38.6, CH	2.97, ddd (9.8, 9.8, 9.8)
9	49.6, CH	2.28, dd (9.1, 6.1)	50.1, CH	2.21, dd (10.0, 6.0)	51.6, CH	2.63, dd (9.8, 8.4)
10	212.8, C		212.7, C		220.7, C	
11	17.5, CH_3_	1.19, d (7.2)	17.6, CH_3_	1.20, d (7.2)	14.5, CH_3_	0.96, d (7.4)
12	201.0, C	9.60, d (1.8)	200.8, C	9.62, d (1.8)	204.9, CH	9.64, d (2.6)
13	139.0, C		138.8, C		136.4, C	
14	22.2, CH_3_	1.68, br s	22.5, CH_3_	1.75, d (0.7)	22.4, CH_3_	1.66, br s
15	125.9, CH	5.29, br t (7.7)	130.9, CH	6.01, br d (11.0)	129.3, CH	5.22, br t (7.0)
16	32.6, CH_2_	2.59, m 2.35, m	126.1, CH	6.59, dd (15.5, 11.0)	28.1, CH_2_	2.84, m 2.73, m
17	78.3, CH	5.12, dd (7.6, 5.2)	138.7, CH	5.76, d (15.5)	124.0, CH	5.09, br t (7.1)
18	144.5, C		82.6, C		132.5, C	
19	113.2, CH_2_	4.94, br s 4.91, dq (1.6, 1.6)	24.6, CH_3_	1.36, s	25.9, CH_3_	1.69, br s
20	18.7, CH_3_	1.76, br s	25.3, CH_3_	1.31, s	17.9, CH_3_	1.64, br s
CH_3_*CO*O (5)	172.2		172.2			
*CH_3_*COO (5)	20.9	1.96, s	20.9	1.96, s		
CH_3_*CO*O (17)	172.1					
*CH_3_*COO (17)	21.0	2.03, s				

^a^^1^H at 500 MHz, ^13^C at 125 MHz; ^b^ assignments aided by COSY, HSQC, HMBC, and NOESY experiments.

**Table 3:** NMR data of ruguloptones A–C (**5**–**7**) in CD_3_OD ^a,b^.

Position	5		6		7	
	δ_C_, Type	δ_H_, m (*J* in Hz)	δ_C_, Type	δ_H_, m (*J* in Hz)	δ_C_, Type	δ_H_, m (*J* in Hz)
1	40.2, CH	2.77, m	42.7, CH	2.67, m	40.3, CH	2.67, m
2	76.8, CH	5.01, d (6.3)	73.4, CH	4.10, d (6.3)	76.7, CH	5.01, d (6.4)
3	41.6, CH_2_	2.56, dd (19.9, 6.3) 2.31, br d (19.9)	44.2, CH_2_	2.43, dd (19.4, 5.8) 2.11, br d (19.4)	41.8, CH_2_	2.57, dd (19.7, 6.4) 2.37, br d (19.7)
4	47.7, CH	3.06, m	47.8, CH	3.06, m	153.8, C	
5	79.8, CH	5.23, br d (4.3)	79.8, CH	5.22, br d (4.7)	78.2, CH	5.63, br dd (7.5, 4.8)
6	35.0, CH_2_	1.96, m 1.76, m	35.2, CH_2_	1.95 ddd (15.0, 9.2, 4.7) 1.76 m	38.0, CH_2_	2.09, ddd (14.5, 7.5, 3.7) 1.98, m
7	39.54^c^, CH	3.65, ddd (11.1, 9.2, 9.2)	39.6, CH_2_	3.65, ddd (11.0, 9.2, 9.2)	41.4, CH	3.51, m
8	39.52^c^, CH	2.65, ddd (12.6, 11.1, 7.7)	39.7, CH	2.64, ddd (12.8, 11.0, 7.7)	43.4, CH	2.87, m
9	50.5, CH	2.86, ddd (12.6, 6.9, 0.9)	49.5, CH	2.99, dd (12.8, 7.0, 0.8)	53.1, CH	2.87, m
10	217.2, C		219.7, C		216.0, C	
11	13.7, CH_3_	0.92, d (7.3)	14.3, CH_3_	0.86, d (7.4)	14.2, CH_3_	0.99, d (7.3)
12	64.0, CH_2_	4.09, dd (10.8, 5.0) 4.03, dd (10.8, 10.8)	64.1, CH_2_	4.09, m 4.04, dd (10.8, 10.8)	113.4, CH_2_	5.27, br s 5.03, br s
13	135.9, C		136.3, C		137.9, C	
14	22.0, CH_3_	1.73, d (1.2)	22.2, CH_3_	1.76, d (1.2)	22.4, CH_3_	1.57, br s
15	130.0, CH	5.23, br t (7.4)	129.6, CH	5.24, br t (7.3)	128.4, CH	5.12, br t (7.2)
16	28.0, CH_2_	2.81, m 2.70, m	28.0, CH_2_	2.83, ddd (16.0, 7.3, 7.3) 2.72, m	27.7, CH_2_	2.76, m 2.67, m
17	123.8, CH	5.05 br t, (7.2)	124.0, CH	5.07, br t (7.1)	123.7, CH	5.05, br t (6.9)
18	132.6, C		132.5, C		132.8, C	
19	25.8, CH_3_	1.68, d (1.1)	25.9, CH_3_	1.68, d (1.1)	25.8, CH_3_	1.68, br s
20	17.8, CH_3_	1.63, br s	17.8, CH_3_	1.63, d (0.6)	17.9, CH_3_	1.63, br s
CH_3_*CO*O (2)	172.2, C				172.1, C	
*CH_3_*COO (2)	20.8 ^d^	2.04 ^c^, s			21.0 ^c^, CH_3_	2.03 ^c^, s
CH_3_*CO*O (5)	172.3, C		172.4, C		172.7, C	
*CH_3_*COO (5)	21.0 ^d^	2.048 ^c^, s	20.8 ^c^, CH_3_	2.04 ^c^, (s)	21.3 ^c^, CH_3_	2.04 ^c^, s
*CH_3_*COO (12)	172.9, C		172.9, C			
*CH_3_*COO (12)	21.3 ^d^	2.045 ^c^, s	21.3 ^c^, CH_3_	2.05 ^c^, (s)		

^a^^1^H at 600 MHz, ^13^C at 150 MHz; ^b^ assignments aided by COSY, HSQC, HMBC, and NOESY experiments; ^c,d^ assignments marked with the same letter in the same column may be interchanged.

**Table 4:** NMR data of ruguloptones D–F (**8**–**10**) in CD_3_OD ^a,b^.

Position	8		9		10	
	δ_C_, Type	δ_H_, m (*J* in Hz)	δ_C_, Type	δ_H_, m (*J* in Hz)	δ_C_, Type	δ_H_, m (*J* in Hz)
1	34.6 ^c^, CH	2.68, m	34.8, CH	2.64, m	42.3, CH	3.05, m
2	28.6, CH_2_	2.05, m 1.66, m	28.8, CH_2_	2.05, m 1.65, m	170.9, CH	7.72, dd (5.8, 2.8)
3	34.5 ^c^, CH_2_	2.25, m 2.09, m	35.0, CH_2_	2.22, m 2.11, dd (9.0, 9.0)	133.2, CH	6.06, dd (5.8, 1.9)
4	141.8, C		142.9, C		47.6, CH	2.76, m
5	130.5, CH	5.76, br s	126.6, CH	5.39, br s	77.6, CH	5.44, ddd (5.8, 5.8, 2.5)
6	37.9, CH_2_	2.57, m 2.05, m	37.9, CH_2_	2.48, m 2.01, m	36.9, CH_2_	2.24, ddd (14.0, 9.2, 5.8) 1.81, ddd (14.0, 7.8, 2.5)
7	43.7, CH	3.68, br t (7.3)	44.2, CH	3.57, ddd (7.5, 7.5, 3.0)	42.6, CH	3.48, m
8	45.9, CH	2.97, m	47.4, CH	2.88, m	40.9, CH	2.32, ddd (9.4, 9.4, 4.4)
9	55.5, CH	2.68, m	55.1, CH	2.57, ddd (8.4, 7.0, 1.3)	49.0, CH	2.54, dd (6.1, 4.4)
10	220.9, C		221.0, C		213.2, C	
11	15.5, CH_3_	0.91, d (6.6)	15.8, CH_3_	0.93, d (7.1)	17.9, CH_3_	1.13, d (7.4)
12	65.0, CH_2_	4.74, d (13.7) 4.67, d (13.7)	16.6, CH_3_	1.68, br s	62.0, CH_2_	3.46, m 3.42, dd (10.7, 4.7)
13	139.2, C		139.7, C		136.5, C	
14	23.1, CH_3_	1.58, br s	23.0, CH_3_	1.58, br s	22.0, CH_3_	1.71, d (1.2)
15	127.4, CH	5.07, br t (7.2)	126.9, CH	5.05, br t (7.6)	129.4, CH	5.35, br t (7.3)
16	27.9, CH_2_	2.80, m 2.68, m	27.9, CH_2_	2.78, m 2.68, m	28.0, CH_2_	2.73, m
17	124.0, CH	5.07, br t (7.2)	124.2, CH	5.07, br t (7.1)	124.0, CH	5.05, br t (7.0)
18	132.5, C		132.3, C		132.5, C	
19	25.9, CH_3_	1.68, br s	25.9, CH_3_	1.68, br s	25.9, CH_3_	1.68, d (1.2)
20	17.8, CH_3_	1.63, br s	17.8, CH_3_	1.62, br s	17.5, CH_3_	1.64, br s
CH_3_*CO*O	172.6, C				172.8, C	
*CH_3_*COO	20.9, CH_3_	2.01, s			21.2, CH_3_	2.05, s

^a^^1^H at 500 MHz, ^13^C at 125 MHz; ^b^ assignments aided by COSY, HSQC, HMBC, and NOESY experiments; ^c^ assignments marked with the same letter in the same column may be interchanged.

## Supplementary Materials

The following are available online at www.mdpi.com/article/10.3390/md19120677/s1: ^1^H and ^13^C NMR spectra of compounds **1**–**10** (Figures S1–S20); and the results of cytotoxicity assays on Bv.2 cells (Figure S21) and RAW 264.7 cells (Figure S22).
