# Supplementary material for: Diterpenoids from the Brown Alga Rugulopteryx okamurae and Their Anti-Inflammatory Activity
Source: Mar Drugs. 2021 Nov 27;19(12):677. doi: 10.3390/md19120677 (PMC8704470; doi:10.3390/md19120677)
Supplement: Supplementary file 1 [file marinedrugs-19-00677-s001.zip › marinedrugs-1472617-supplementary.pdf]

Supplementary material

Diterpenoids from the Brown Alga *Rugulopteryx okamurae* and their Anti-inflammatory Activity

Belén Cuevas[1,2], Ana I. Arroba[2,3], Carolina de los Reyes[1], Laura Gómez-Jaramillo[2], Mª Carmen González-Montelongo[2] and Eva Zubía [1,*]

[1] Departamento de Química Orgánica, Facultad de Ciencias del Mar y Ambientales, Universidad de Cádiz, 11510-Puerto Real (Cádiz), Spain; belen.cuevas@inibica.es (B.C.), carolina.dereyes@uca.es (C.D.L.R.), eva.zubia@uca.es (E.Z.)

[2] Unidad de Investigación, Instituto de Investigación e Innovación Biomédica de Cádiz (INiBICA), Hospital Universitario Puerta del Mar, Avda. Ana de Viya 21, 11009-Cádiz, Spain; anaarroba@gmail.com (A.I.A.), laugomjar@gmail.com (L.G.J), mcmontel@gmail.com (M.C.G.M)

[3] Departamento de Endocrinología y Nutrición, Hospital Universitario Puerta del Mar, Ana de Viya 21, 11009-Cádiz, Spain.

*Correspondence: eva.zubia@uca.es

Figure S1. ^{1}H NMR spectrum (500 MHz, CD_3OD) of rugukadiol A (**1**)

Figure S2. ^{13}C NMR spectrum (125 MHz, CD_3OD) of rugukadiol A (**1**)

Figure S3. ^{1}H NMR spectrum (500 MHz, CD_3OD) of rugukamural A (**2**)

Figure S4. ^{13}C NMR spectrum (125 MHz, CD_3OD) of rugukamural A (**2**)

Figure S5. ^{1}H NMR spectrum (500 MHz, CD_3OD) of rugukamural B (**3**)

Figure S6. ^{13}C NMR spectrum (125 MHz, CD_3OD) of rugukamural B (**3**)

Figure S7. ^{1}H NMR spectrum (500 MHz, CD_3OD) of rugukamural C (**4**)

Figure S8. ^{13}C NMR spectrum (125 MHz, CD_3OD) of rugukamural C (**4**)

Figure S9. ^{1}H NMR spectrum (500 MHz, CD_3OD) of ruguloptone A (**5**)

Figure S10. ^{13}C NMR spectrum (125 MHz, CD_3OD) of ruguloptone A (**5**)

Figure S11. ^{1}H NMR spectrum (500 MHz, CD_3OD) of ruguloptone B (**6**)

Figure S12. ^{13}C NMR spectrum (125 MHz, CD_3OD) of ruguloptone B (**6**)

Figure S13. ^{1}H NMR spectrum (500 MHz, CD_3OD) of ruguloptone C (**7**)

Figure S14. ^{13}C NMR spectrum (125 MHz, CD_3OD) of ruguloptone C (**7**)

Figure S15. ^{1}H NMR spectrum (500 MHz, CD_3OD) of ruguloptone D (**8**)

Figure S16. ^{13}C NMR spectrum (125 MHz, CD_3OD) of ruguloptone D (**8**)

Figure S17. ^{1}H NMR spectrum (500 MHz, CD_3OD) of ruguloptone E (**9**)

Figure S18. ^{13}C NMR spectrum (125 MHz, CD_3OD) of ruguloptone E (**9**)

Figure S19. ^{1}H NMR spectrum (500 MHz, CD_3OD) of ruguloptone F (**10**)

Figure S20. ^{13}C NMR spectrum (125 MHz, CD_3OD) of ruguloptone F (**10**)

Figure S21. Cell survival Bv.2 cells

Figure S22. Cell survival RAW 246.7 cells

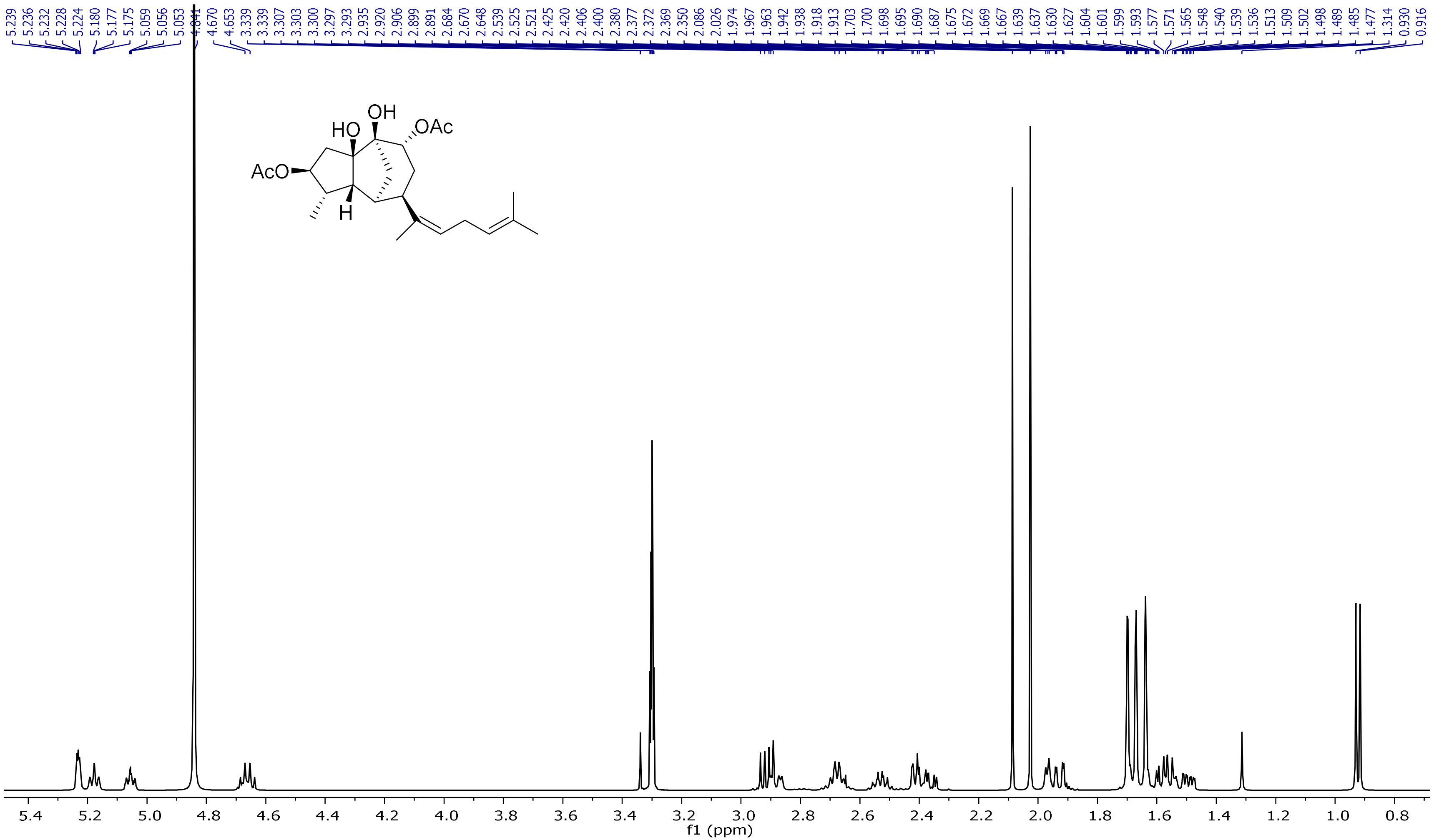

Figure S1. ^{1}H NMR spectrum (500 MHz, CD_3OD) of rugukadiol A (**1**)

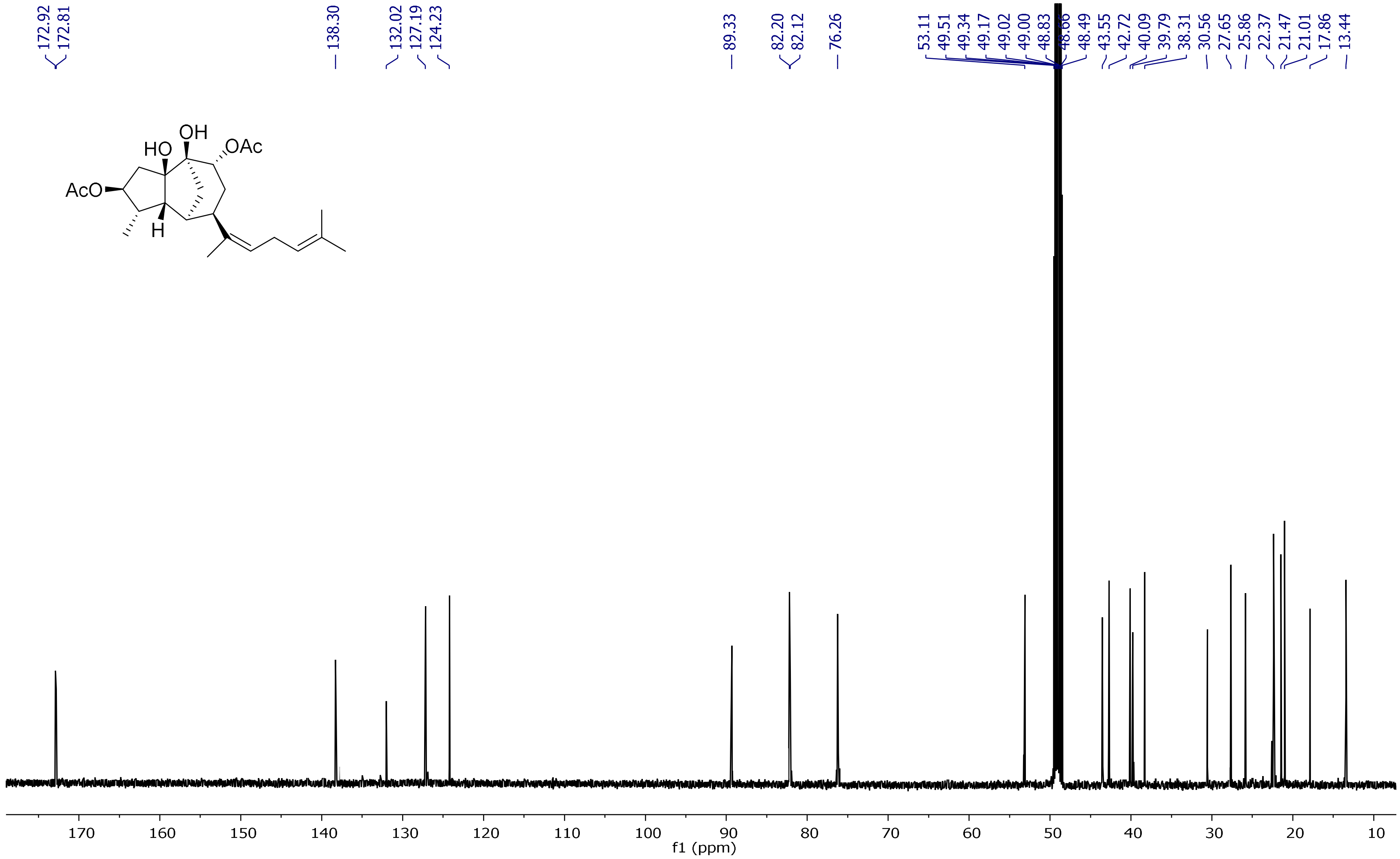

Figure S2. ^{13}C NMR spectrum (125 MHz, CD_3OD) of rugukadiol A (**1**)

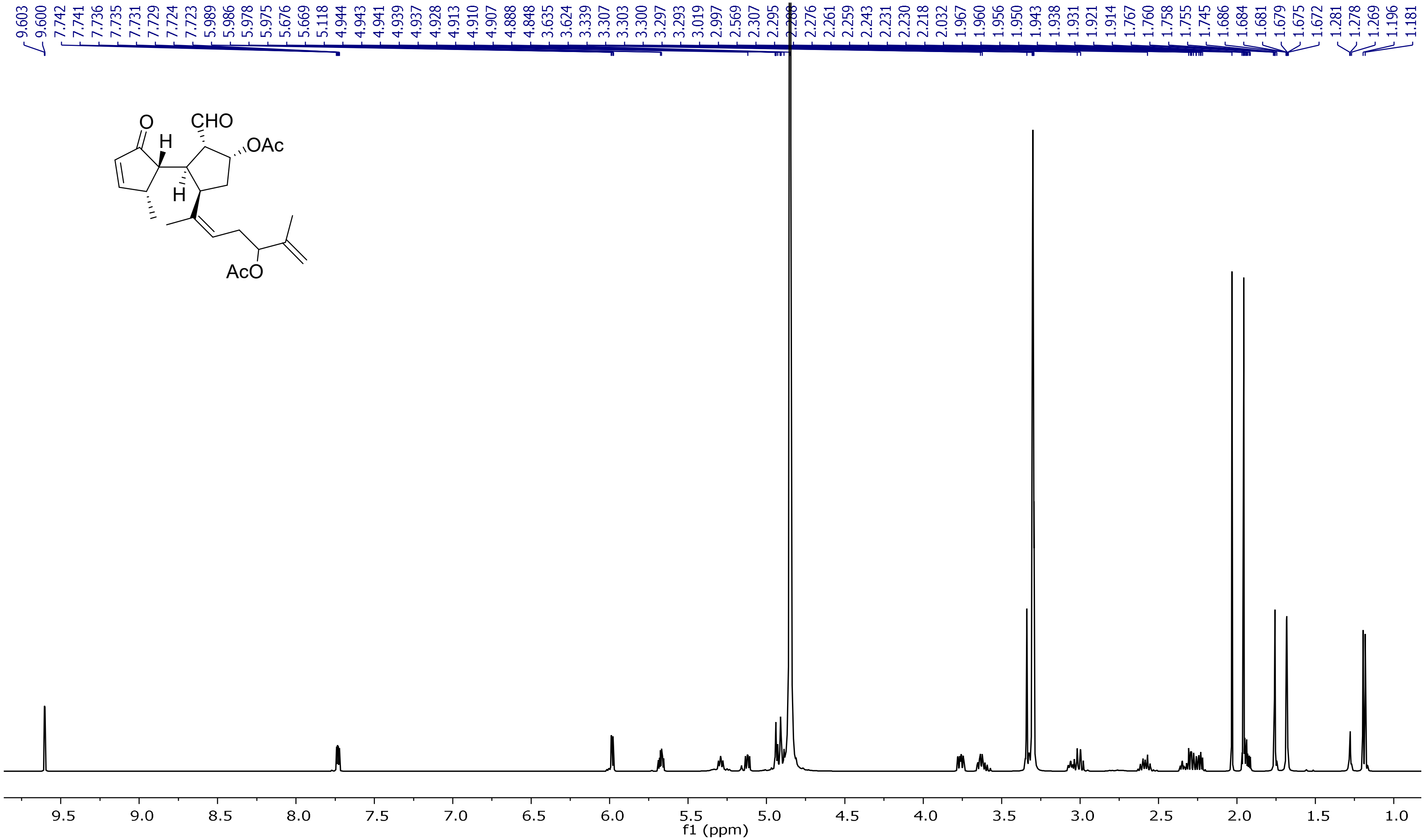

Figure S3. ^{1}H NMR spectrum (500 MHz, CD_3OD) of rugukamural A (**2**)

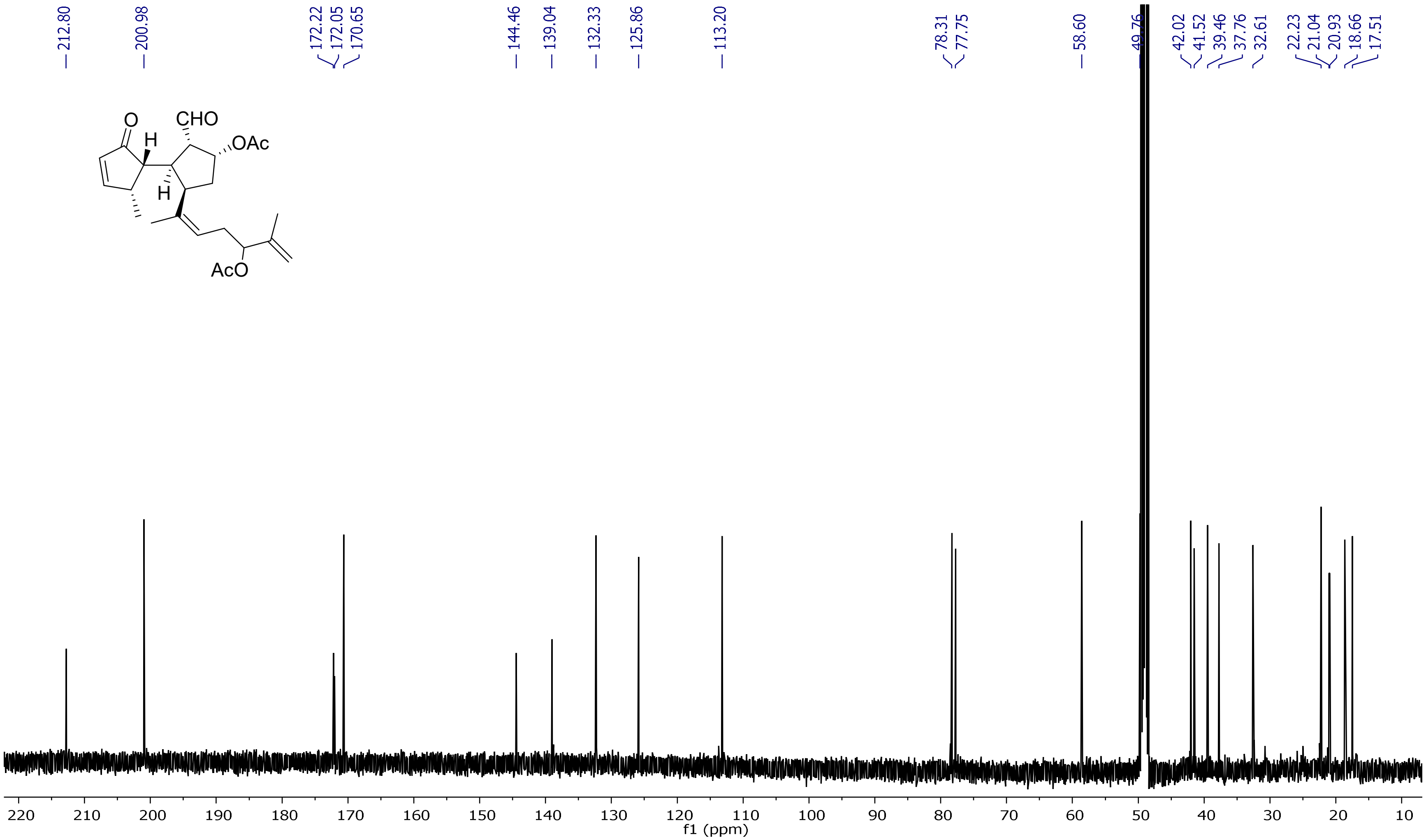

Figure S4. ^{13}C NMR spectrum (125 MHz, CD_3OD) of rugukamural A (**2**)

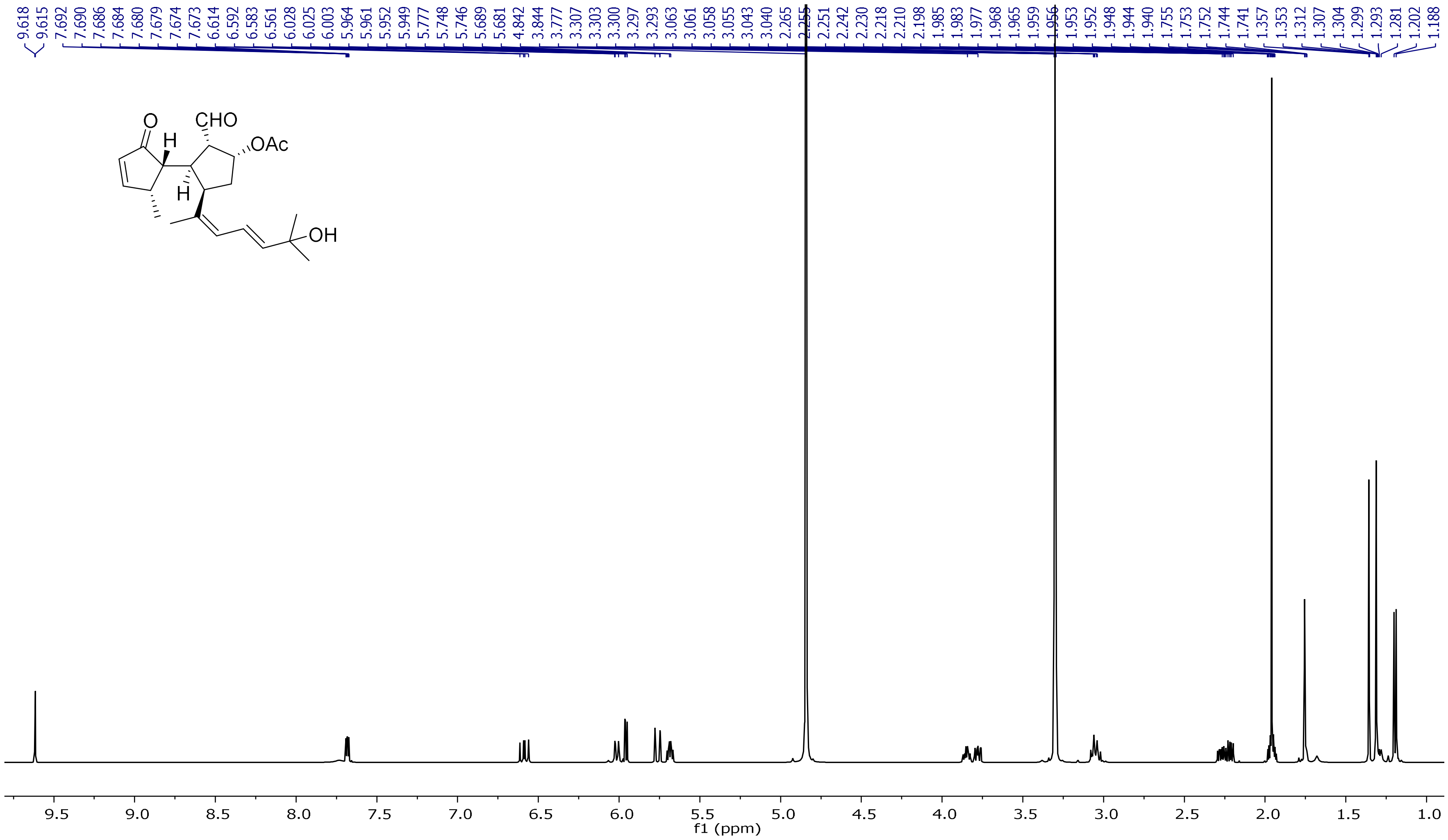

Figure S5. ^{1}H NMR spectrum (500 MHz, CD_3OD) of rugukamural B (**3**)

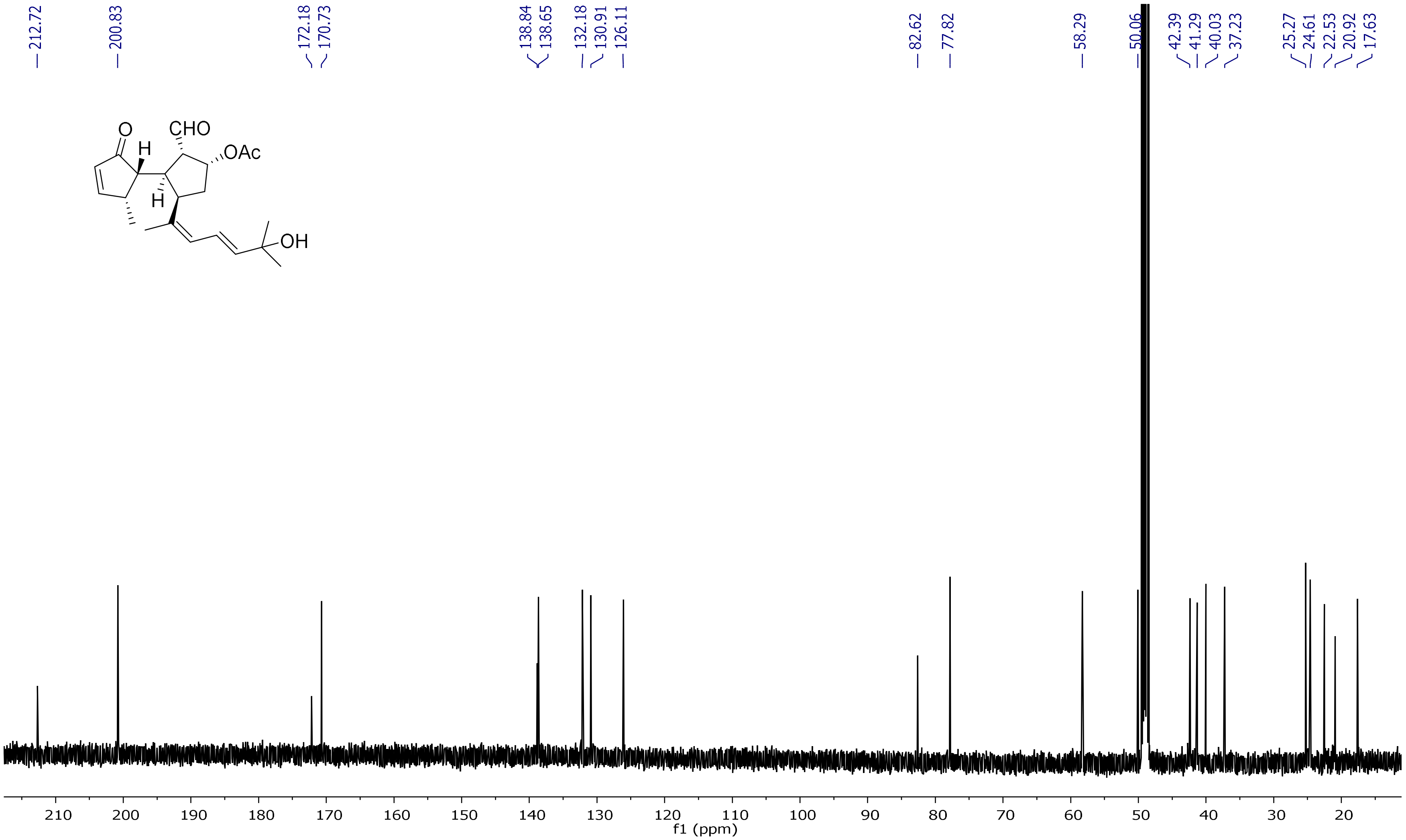

Figure S6. ^{13}C NMR spectrum (125 MHz, CD_3OD) of rugukamural B (**3**)

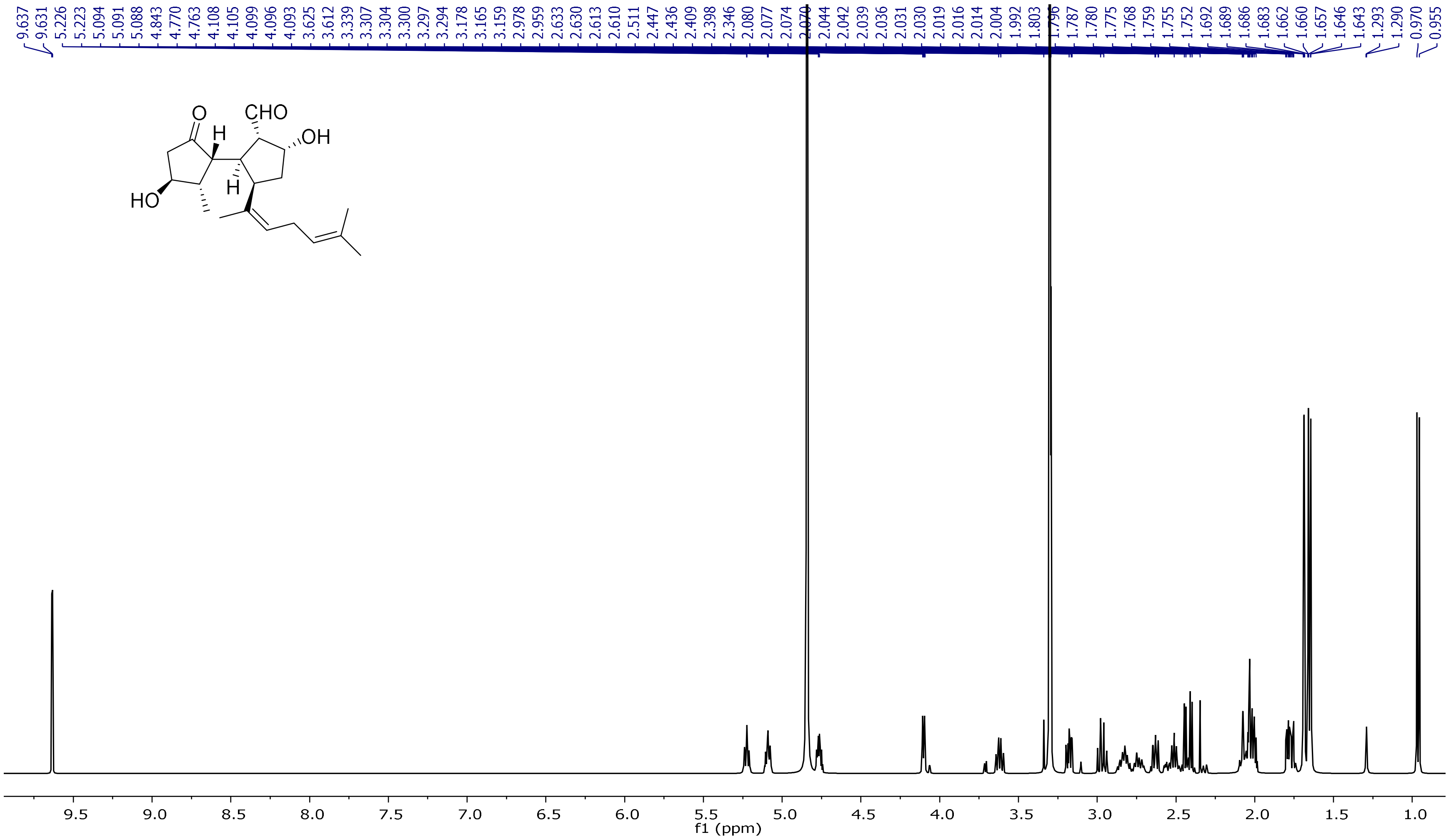

Figure S7. ^{1}H NMR spectrum (500 MHz, CD_3OD) of rugukamural C (**4**)

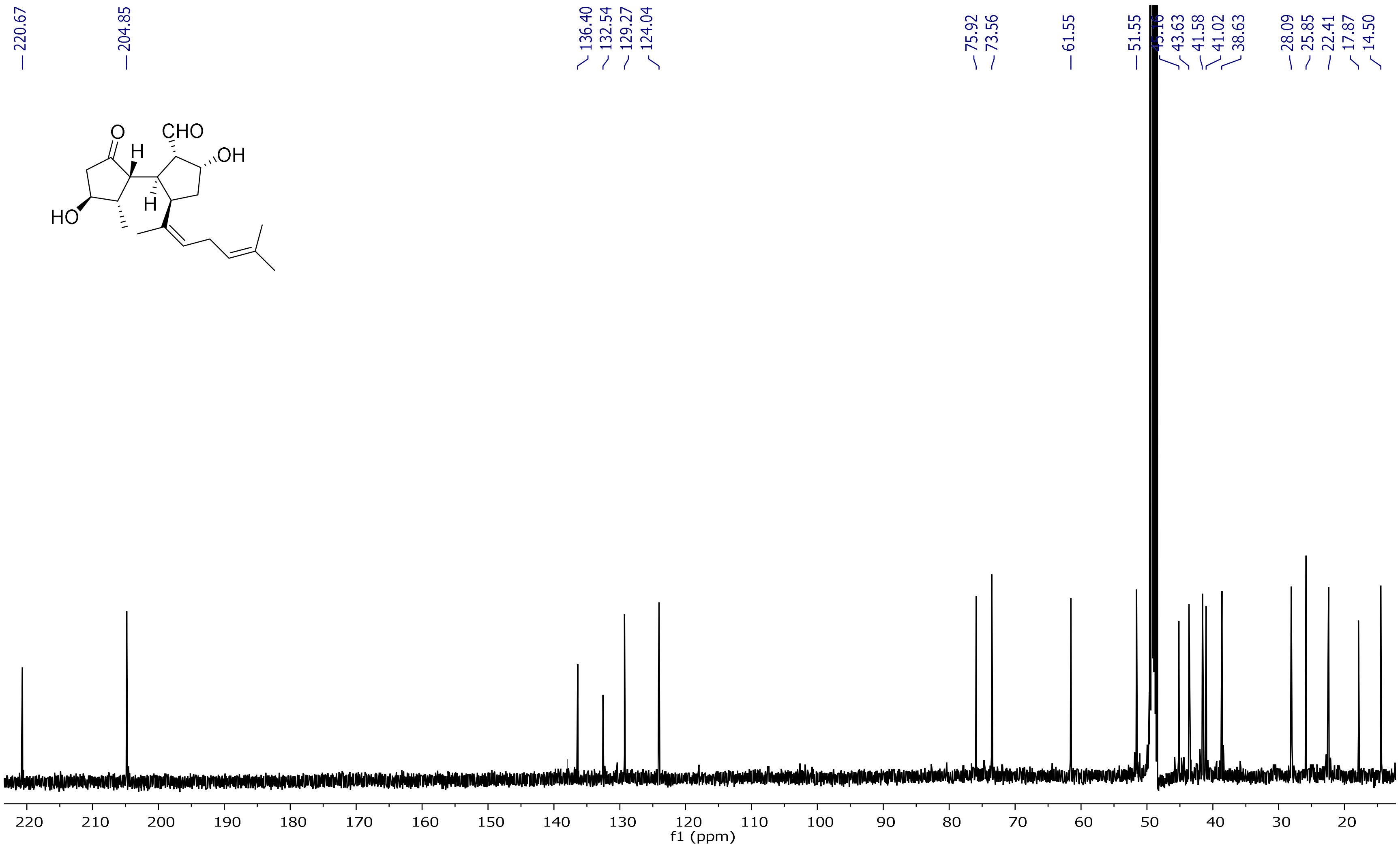

Figure S8. ^{13}C NMR spectrum (125 MHz, CD_3OD) of rugukamural C (**4**)

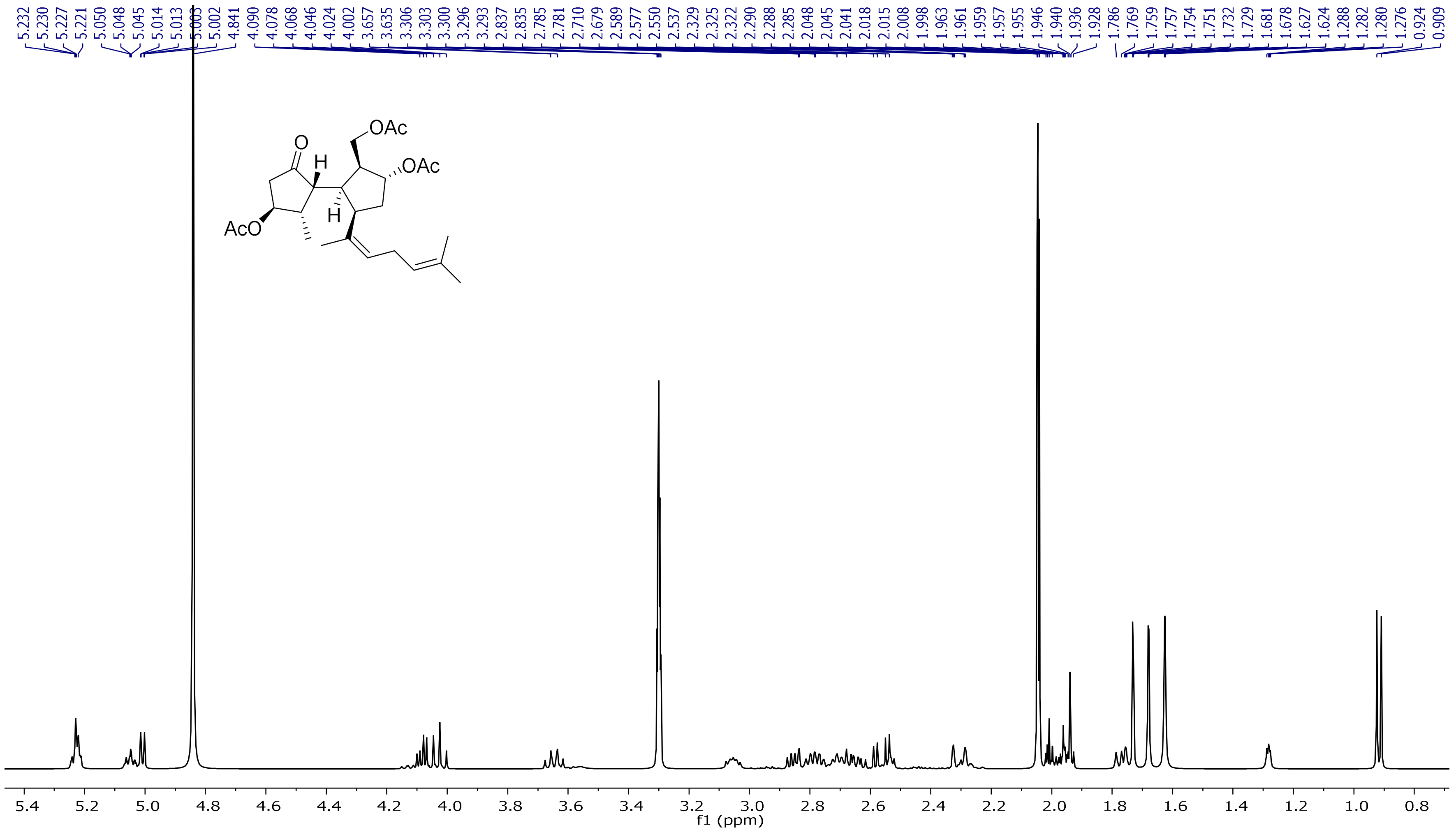

Figure S9. ^{1}H NMR spectrum (500 MHz, CD_3OD) of ruguloptone A (**5**)

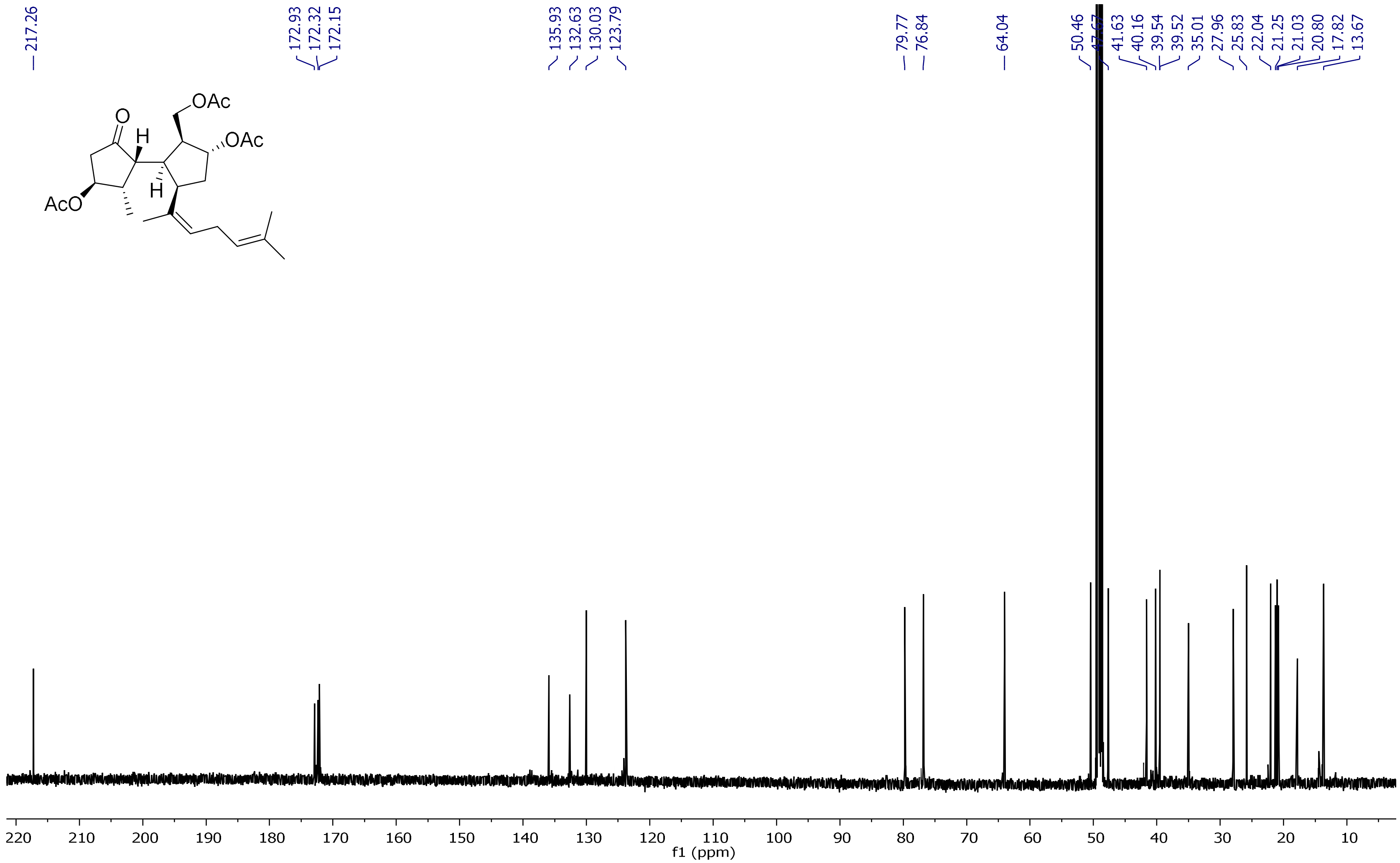

Figure S10. ^{13}C NMR spectrum (125 MHz, CD_3OD) of ruguloptone A (**5**)

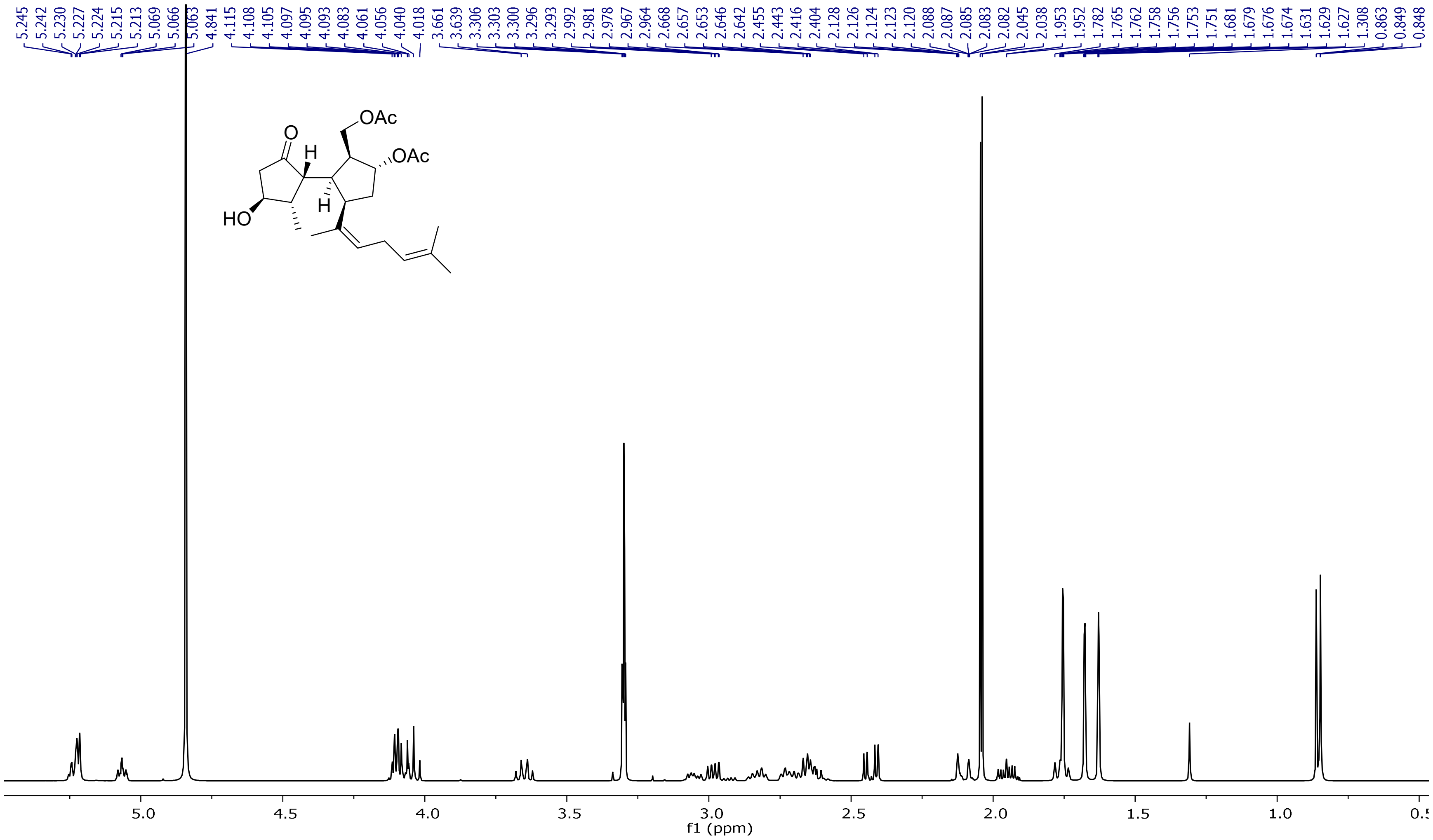

Figure S11. ^{1}H NMR spectrum (500 MHz, CD_3OD) of ruguloptone B (**6**)

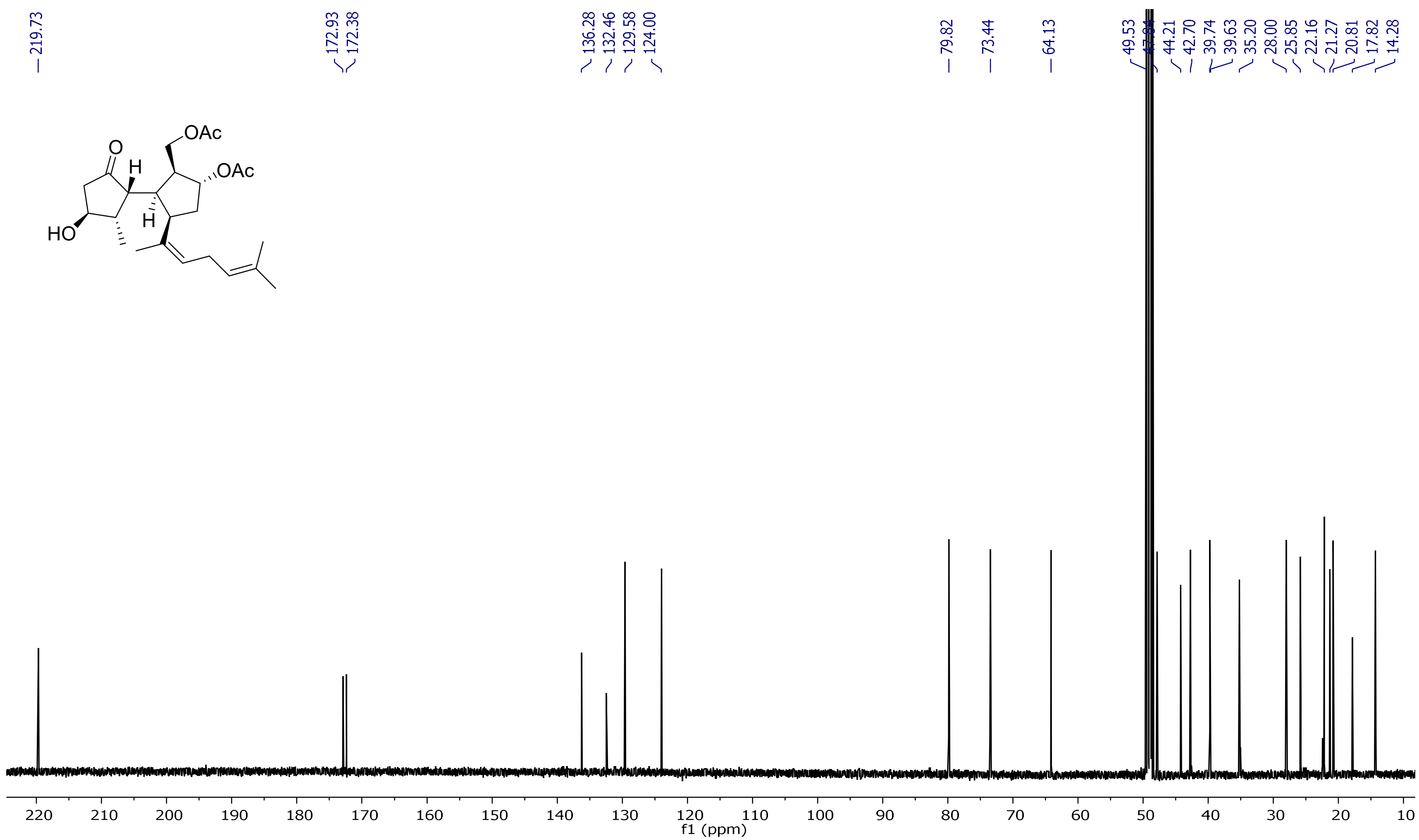

Figure S12. ^{13}C NMR spectrum (125 MHz, CD_3OD) of ruguloptone B (**6**)

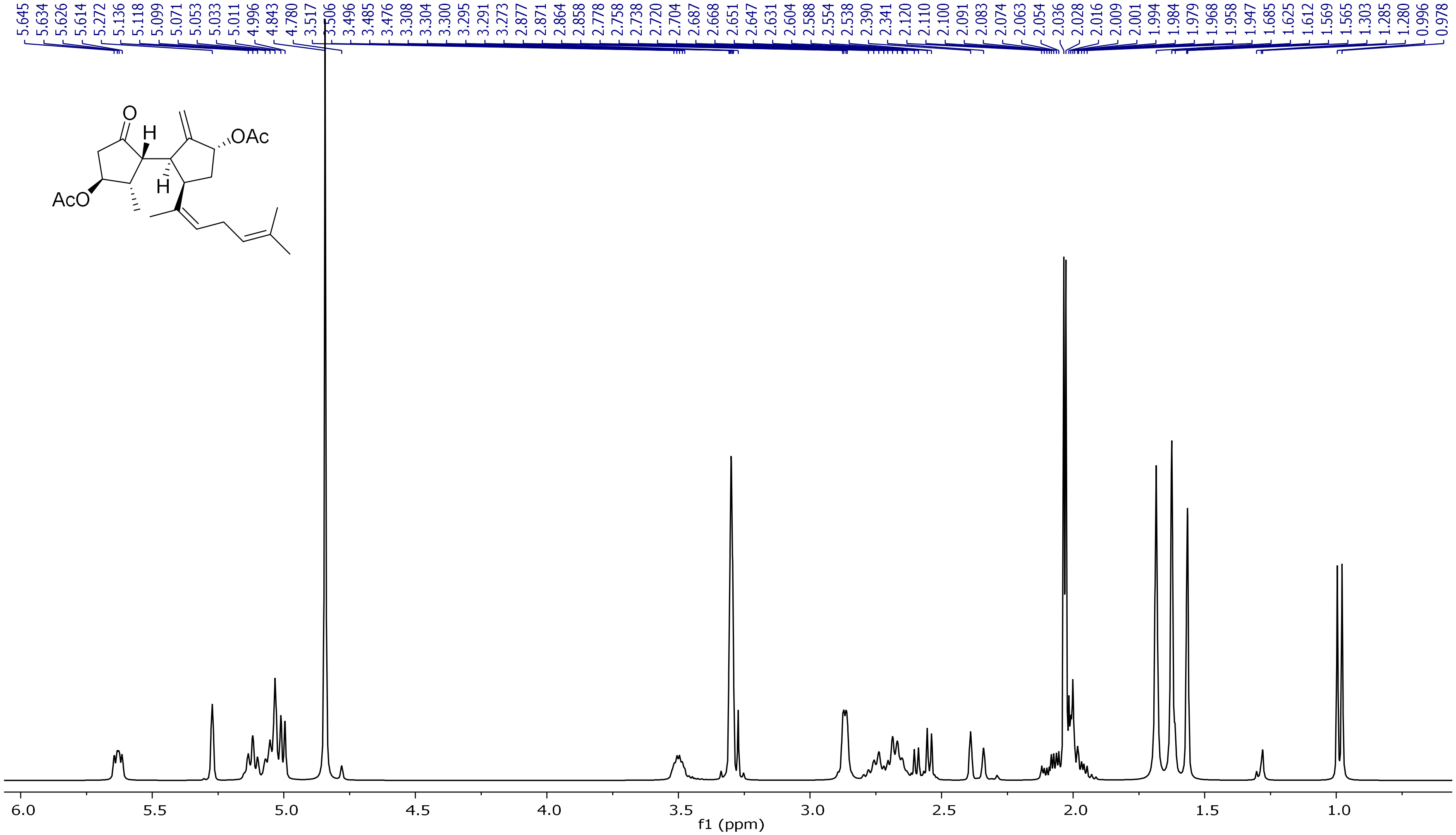

Figure S13. ^{1}H NMR spectrum (500 MHz, CD_3OD) of ruguloptone C (**7**)

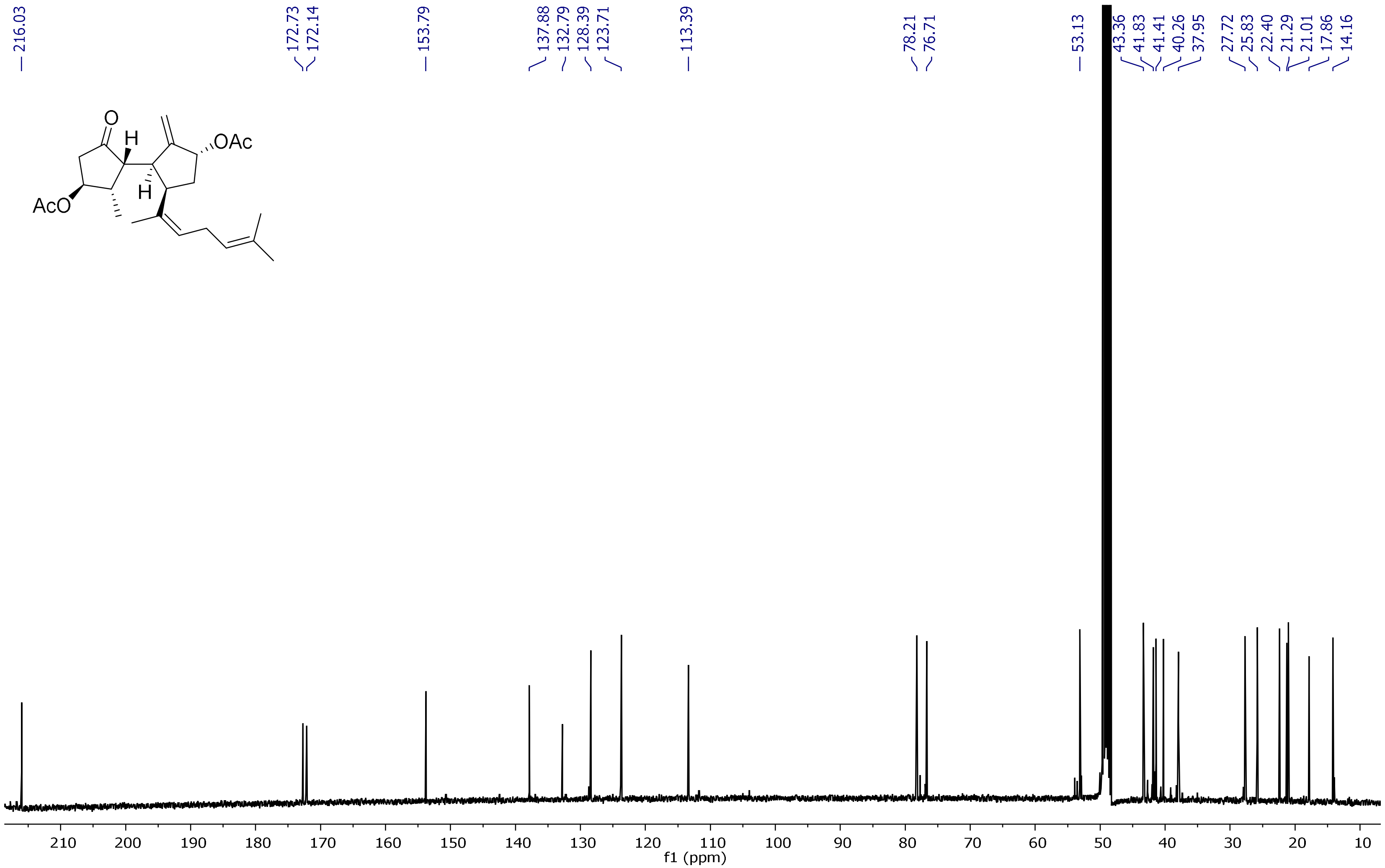

Figure S14. ^{13}C NMR spectrum (125 MHz, CD_3OD) of ruguloptone C (**7**)

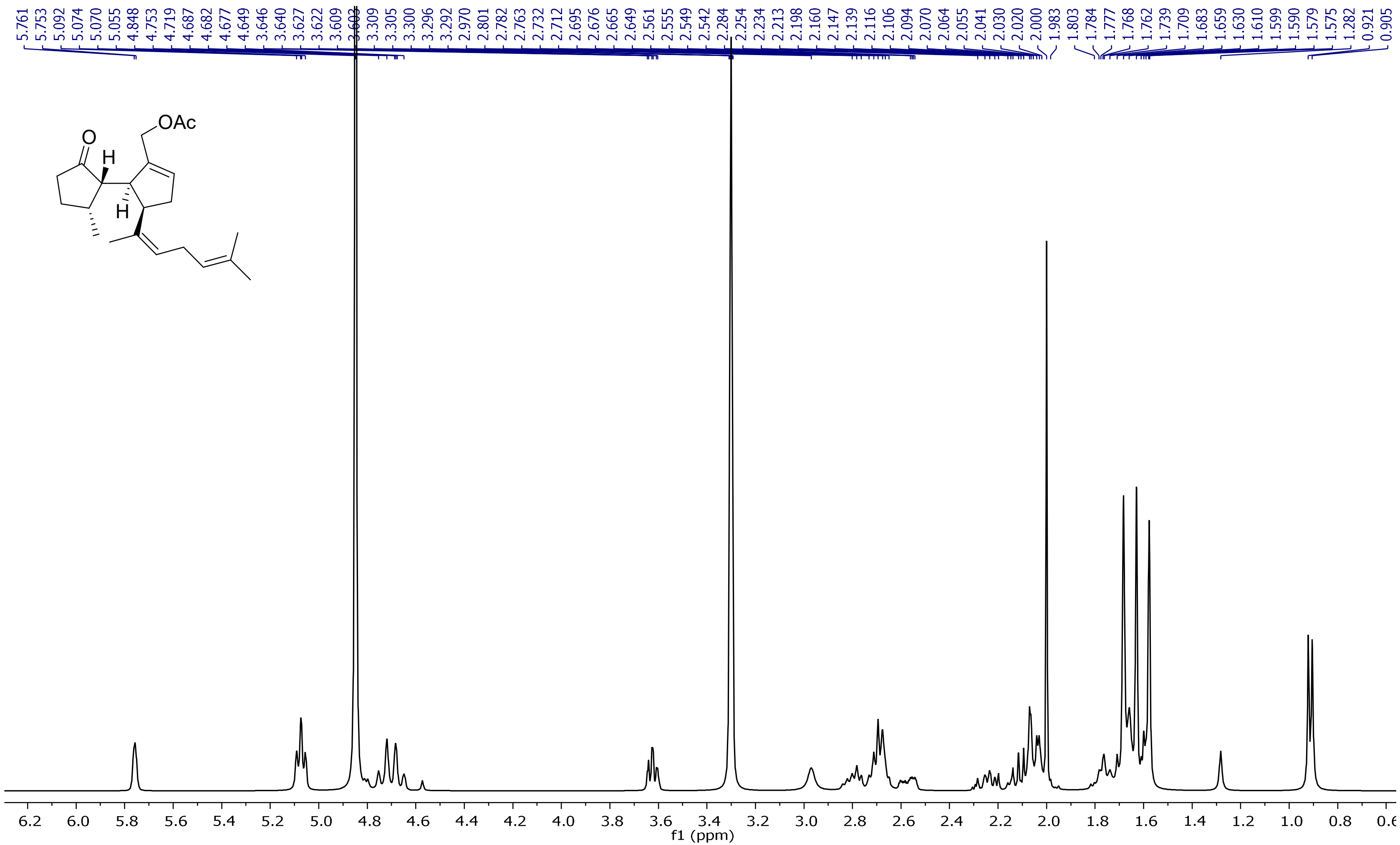

Figure S15. ^{1}H NMR spectrum (500 MHz, CD_3OD) of ruguloptone D (**8**)

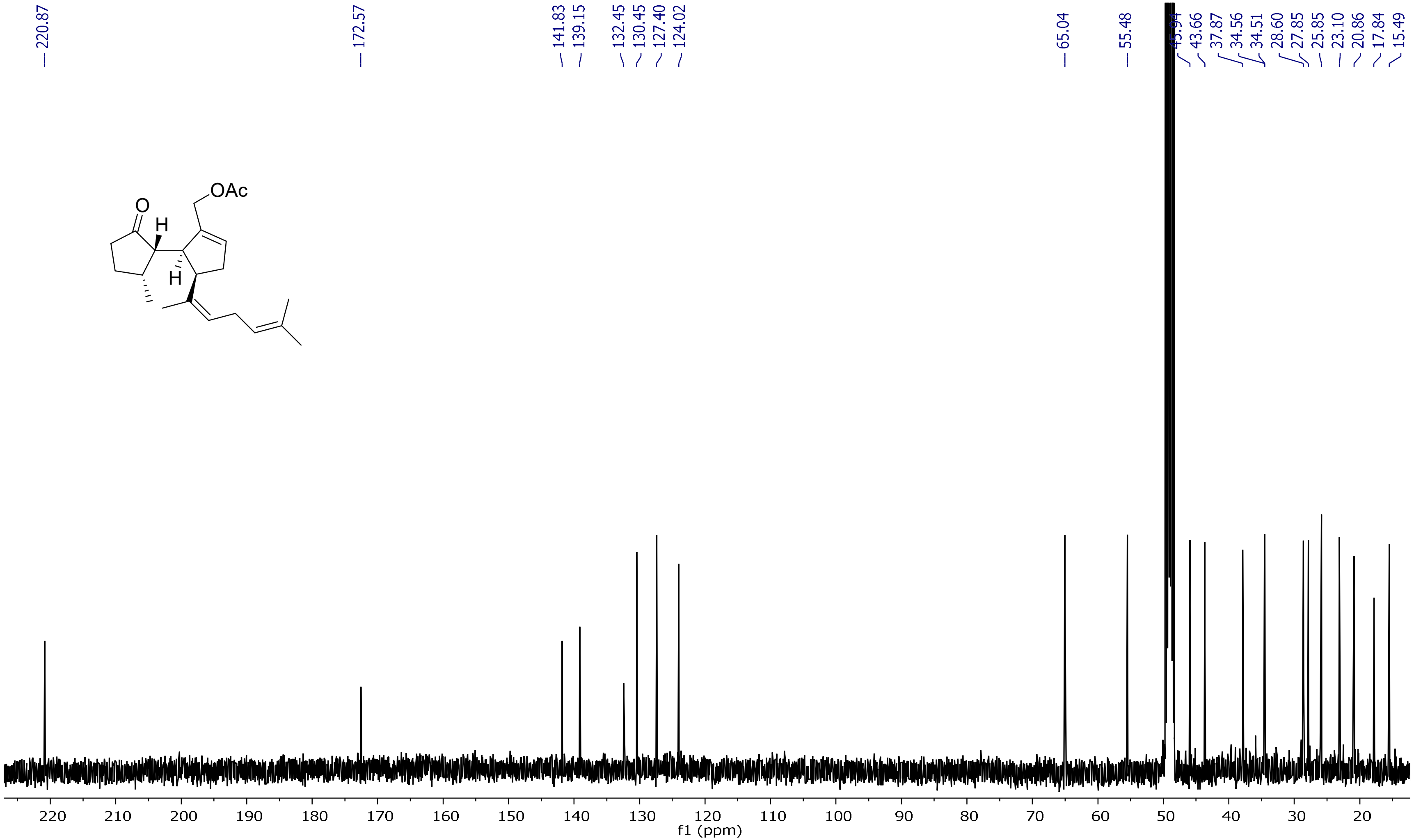

Figure S16. ^{13}C NMR spectrum (125 MHz, CD_3OD) of ruguloptone B (**8**)

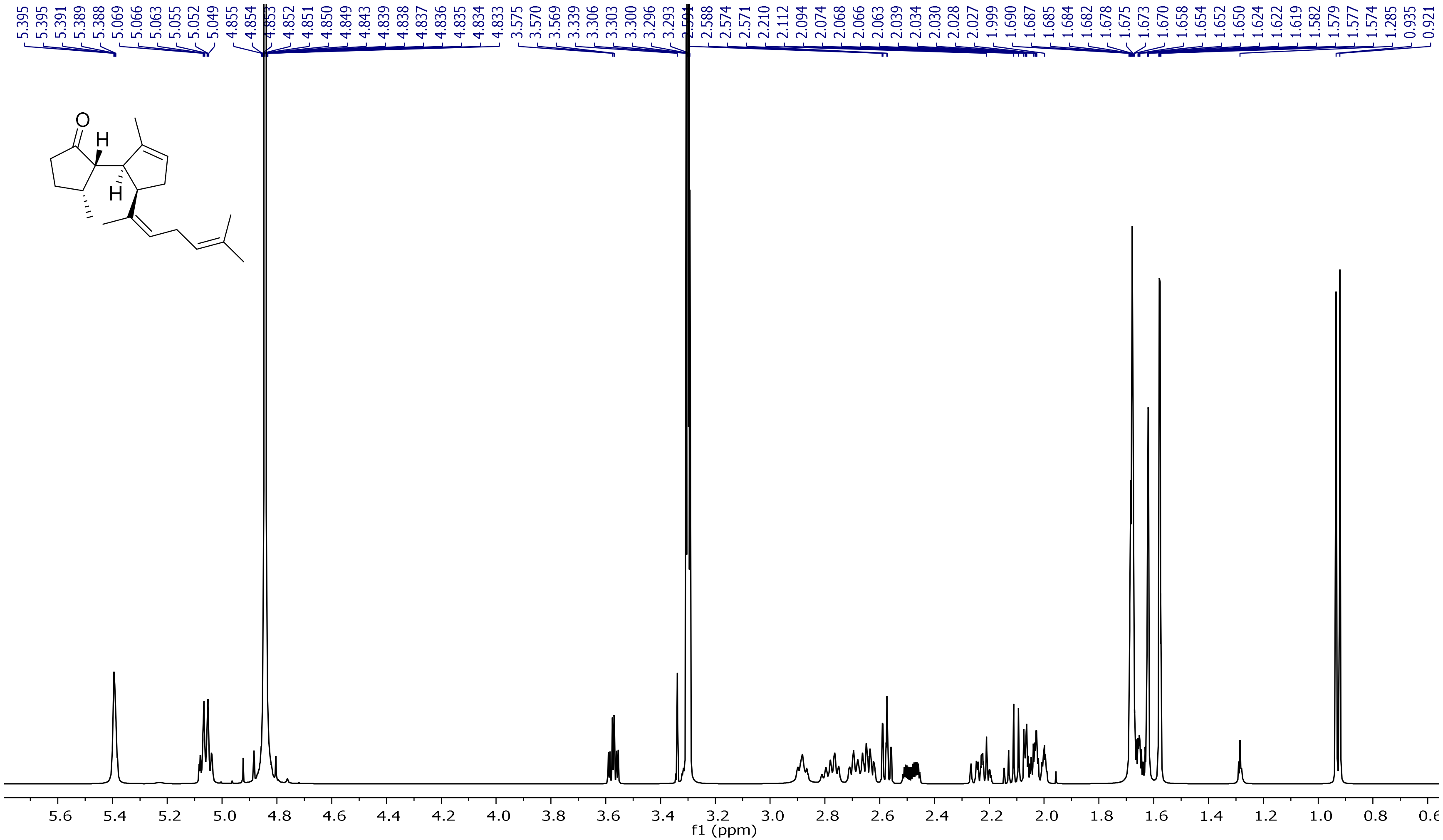

Figure S17. ^{1}H NMR spectrum (500 MHz, CD_3OD) of ruguloptone E (**9**)

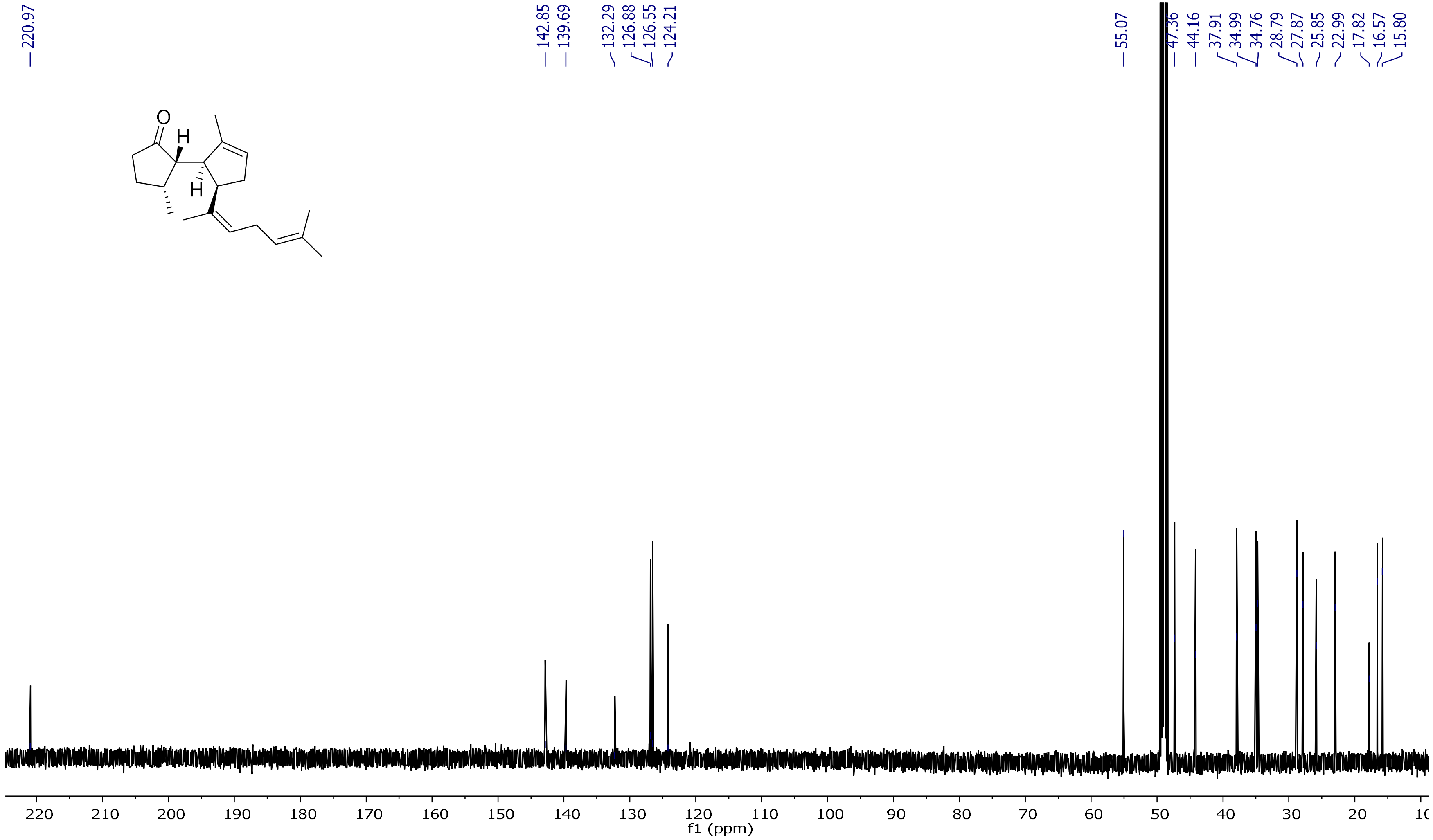

Figure S18. ^{13}C NMR spectrum (125 MHz, CD_3OD) of ruguloptone E (**9**)

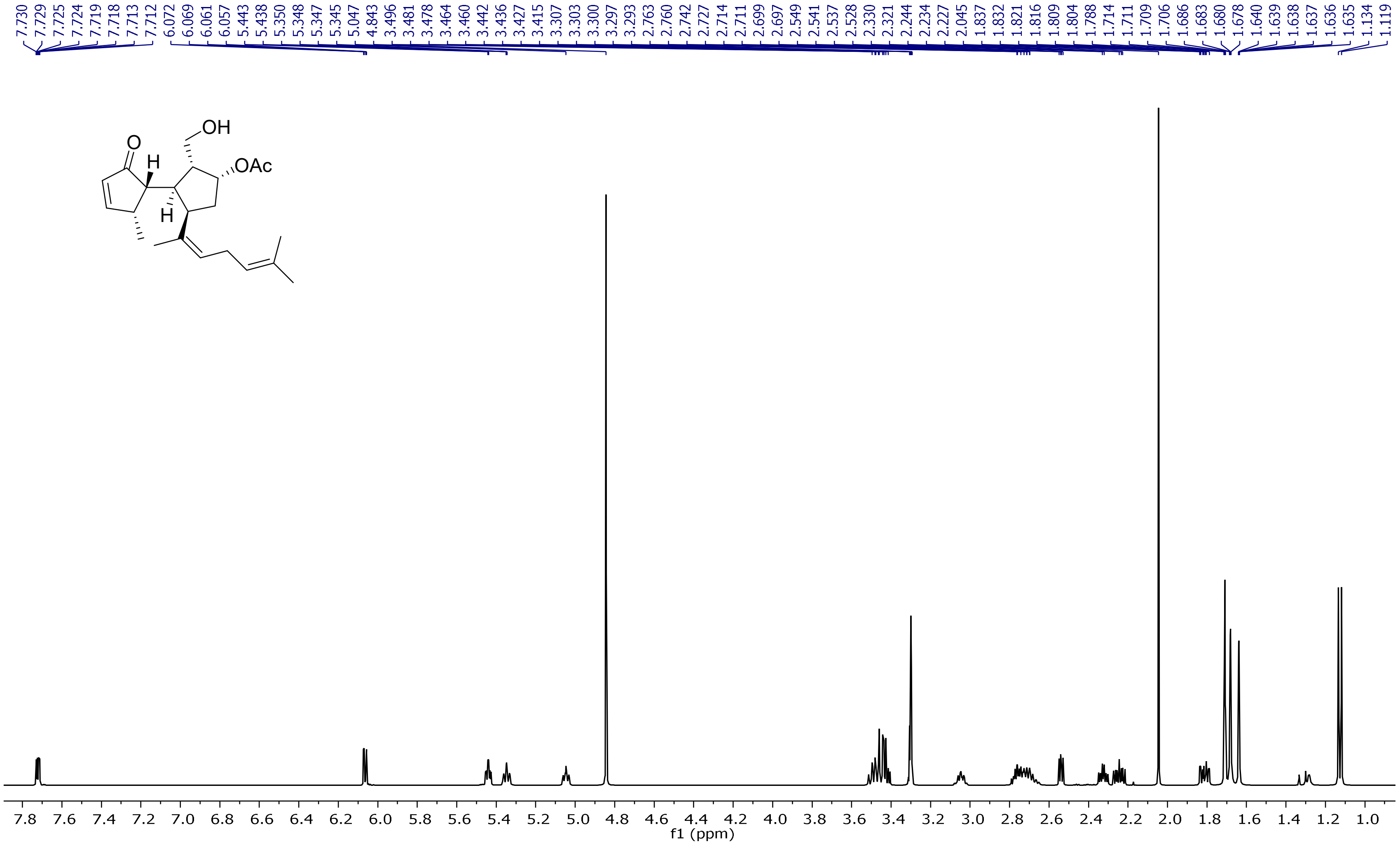

Figure S19. ^{1}H NMR spectrum (500 MHz, CD_3OD) of ruguloptone F (**10**)

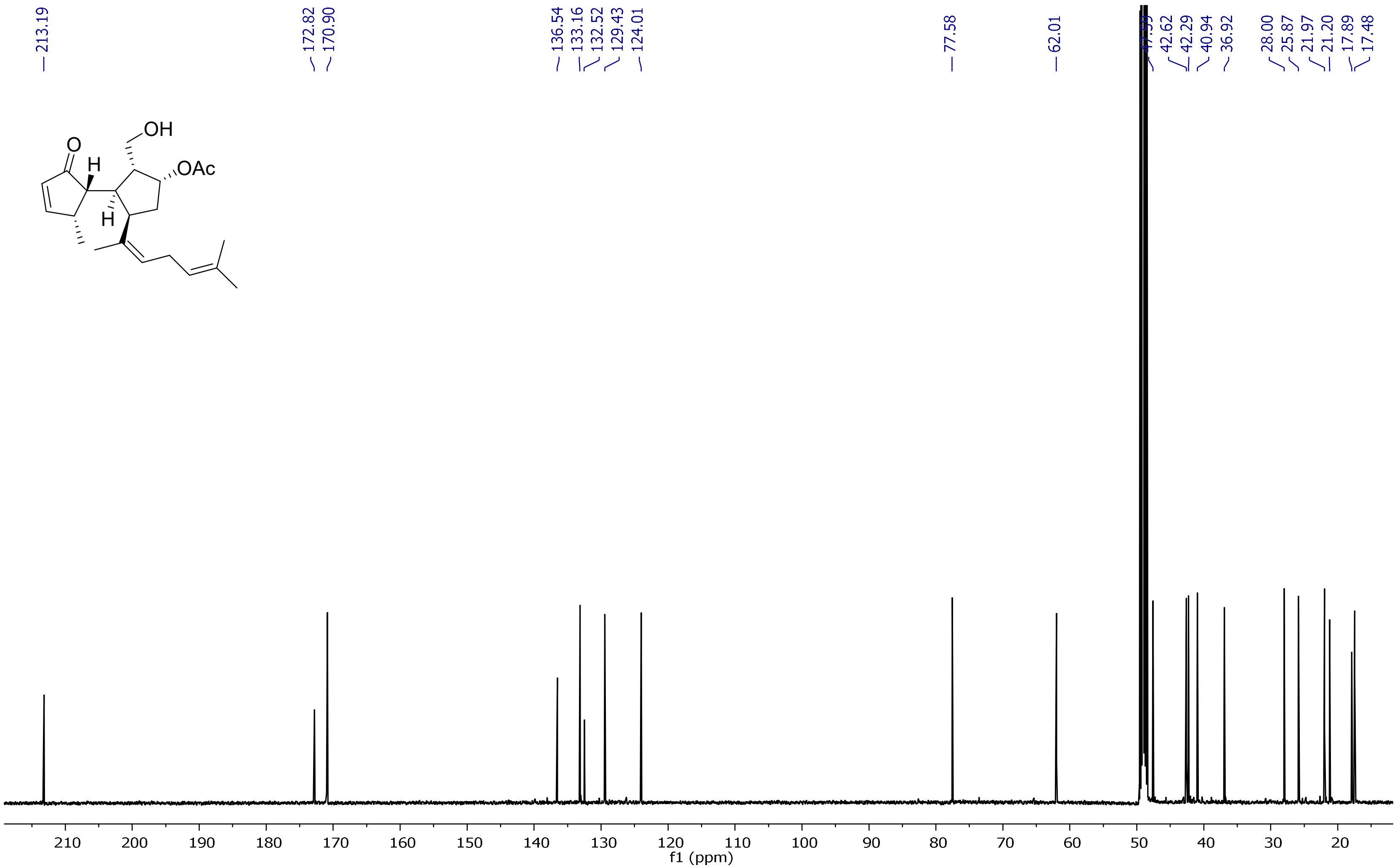

Figure S20. ^{13}C NMR spectrum (125 MHz, CD_3OD) of ruguloptone F (**10**)

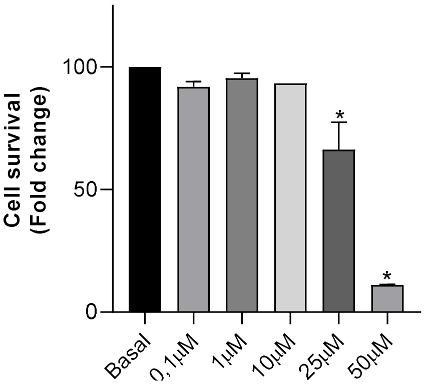

Compound **1**

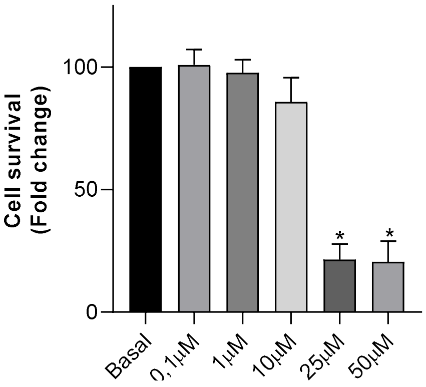

Compound **4**

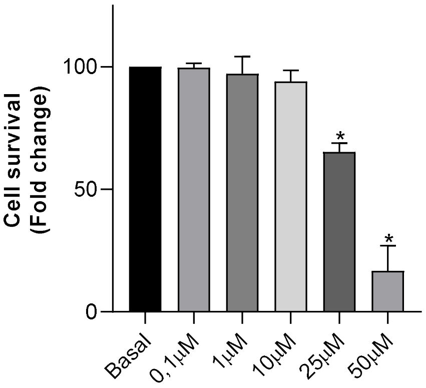

Compound **5**

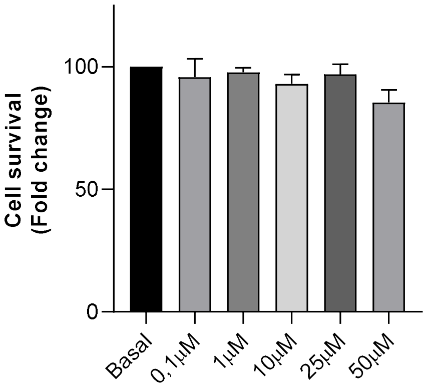

Compound **6**

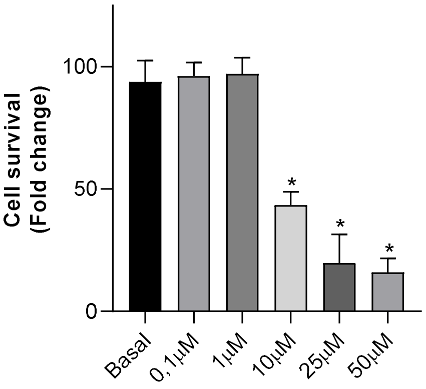

Compound **7**

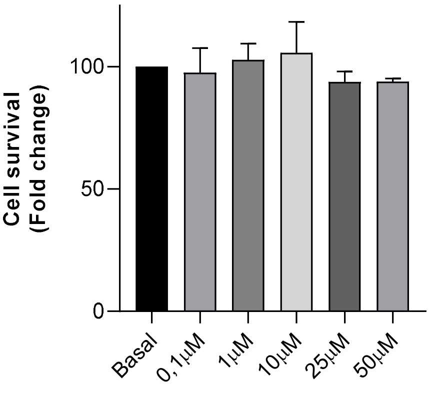

Compound **9**

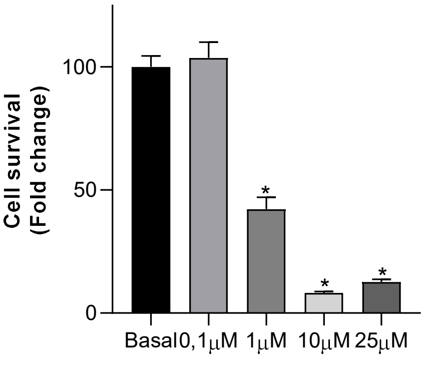

Compound **11**

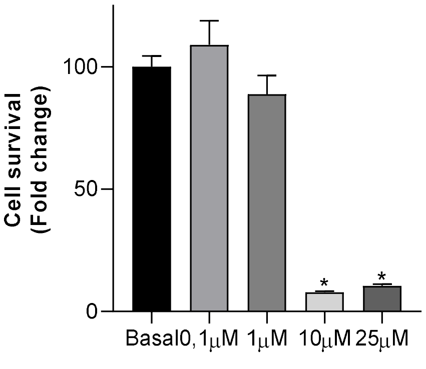

Compound **12**

Figure **S21.** Dose-response cell viability of Bv.2 cells was determined by cristal violet assay. Colorimetric quantification was performed, and the results are mean ± SD (n ≥ 3 independent experiments performed in duplicate). Significant differences were determined by two way ANOVA followed by Bonferroni t-test *$p \leq 0.05$ vs Basal.

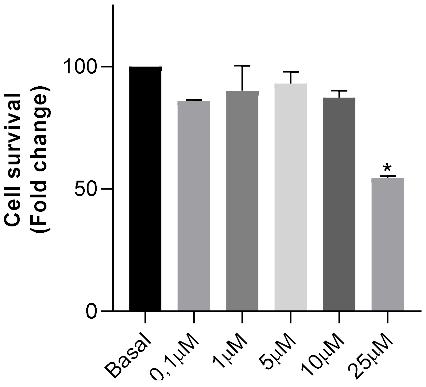

Compound **1**

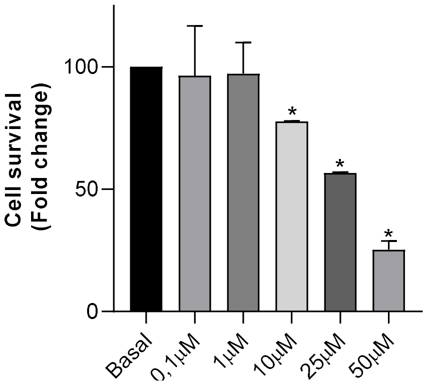

Compound **4**

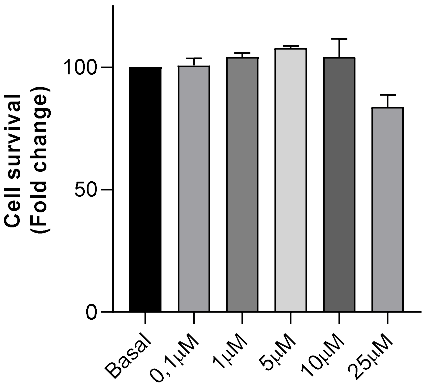

Compound **5**

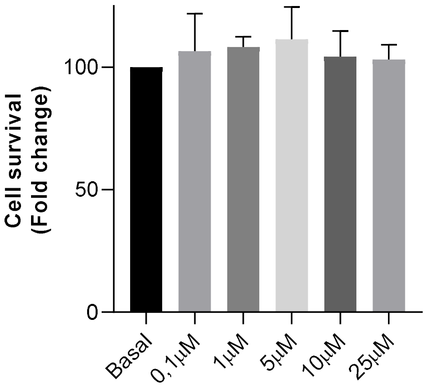

Compound **6**

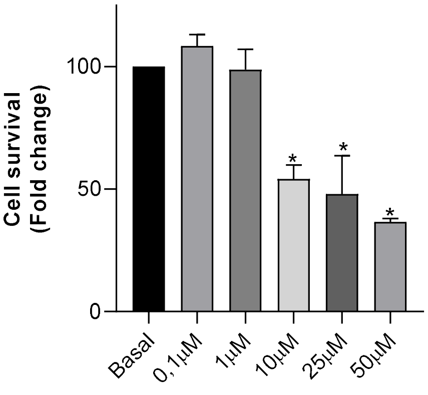

Compound **7**

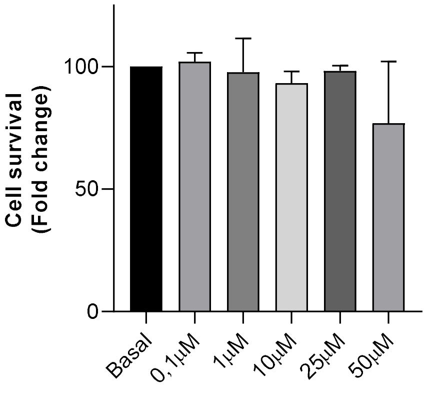

Compound **9**

Figure **S22.** Dose-response cell viability of RAW 264.7 cells was determined by cristal violet assay. Colorimetric quantification was performed, and the results are mean ± SD (n ≥ 3 independent experiments performed in duplicate). Significant differences were determined by two way ANOVA followed by Bonferroni t-test *$p \leq 0.05$ vs Basal.
